# Efficient genome editing in grapevine using CRISPR/LbCas12a system

**DOI:** 10.1186/s43897-023-00069-w

**Published:** 2023-10-18

**Authors:** Chong Ren, Elias Kirabi Gathunga, Xue Li, Huayang Li, Junhua Kong, Zhanwu Dai, Zhenchang Liang

**Affiliations:** 1 State Key Laboratory of Plant Diversity and Specialty Crops, Beijing Key Laboratory of Grape Sciences and Enology, Institute of Botany, the Chinese Academy of Sciences, Beijing, 100093 PR China; 2 China National Botanical Garden, Beijing, 100093 PR China; 3 University of Chinese Academy of Sciences, Beijing, 100049 PR China

**Keywords:** *Vitis vinifera*, CRISPR, LbCas12a, Multiplex editing, crRNA length, Flavonoid

## Abstract

Clustered regularly interspaced short palindromic repeats (CRISPR) /Cas12a system, also known as CRISPR/Cpf1, has been successfully harnessed for genome engineering in many plants, but not in grapevine yet. Here we developed and demonstrated the efficacy of CRISPR/Cas12a from *Lachnospiraceae bacterium ND2006* (LbCas12a) in inducing targeted mutagenesis by targeting the *tonoplastic monosaccharide transporter1* (*TMT1*) and *dihydroflavonol-4-reductase 1* (*DFR1*) genes in 41B cells. Knockout of *DFR1* gene altered flavonoid accumulation in *dfr1* mutant cells. Heat treatment (34℃) improved the editing efficiencies of CRISPR/LbCas12a system, and the editing efficiencies of *TMT1*-crRNA1 and *TMT1*-crRNA2 increased from 35.3% to 44.6% and 29.9% to 37.3% after heat treatment, respectively. Moreover, the sequences of crRNAs were found to be predominant factor affecting editing efficiencies irrespective of the positions within the crRNA array designed for multiplex genome editing. In addition, genome editing with truncated crRNAs (trucrRNAs) showed that trucrRNAs with 20 nt guide sequences were as effective as original crRNAs with 24 nt guides in generating targeted mutagenesis, whereas trucrRNAs with shorter regions of target complementarity ≤ 18 nt in length may not induce detectable mutations in 41B cells. All these results provide evidence for further applications of CRISPR/LbCas12a system in grapevine as a powerful tool for genome engineering.

**Graphical Abstract:**

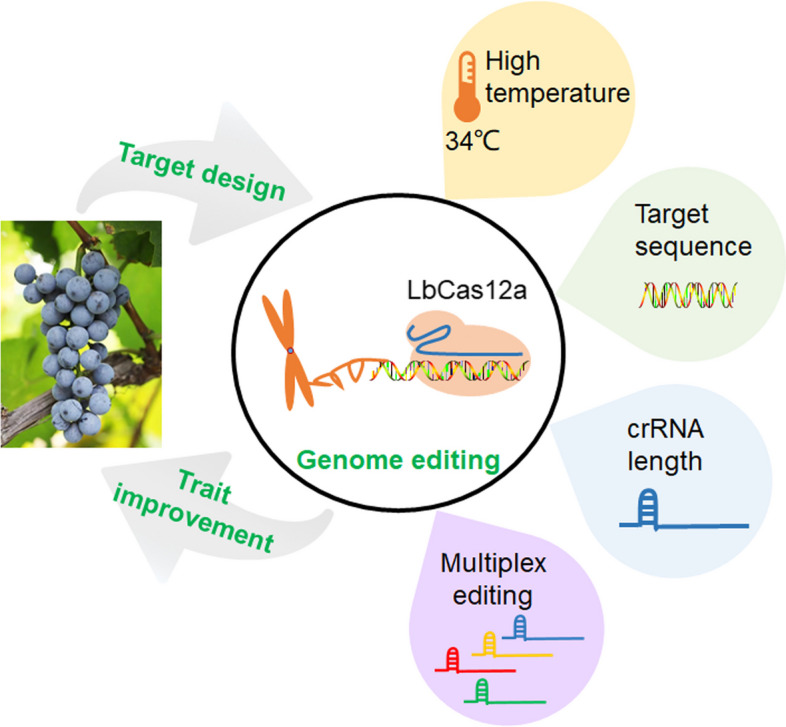

**Supplementary Information:**

The online version contains supplementary material available at 10.1186/s43897-023-00069-w.

## Core

The CRISPR/LbCpf1 system was developed for efficient grape genome editing, and heat treatment, crRNA sequences and length were found to affect editing efficiencies during multiplex genome editing.

## Gene & accession numbers

Sequence data from this article can be found in the database of Grape RNA under the accession numbers: PDS1 (VIT_09s0002g00100), TMT1 (VIT_18s0122g00850), TMT2 (VIT_03s0038g03940), and DFR1 (VIT_18s0001g12800).

## Introduction

The advent of CRISPR (clustered regularly interspaced short palindromic repeats)/Cas (CRISPR-associated protein) systems have revolutionized genome editing in both animals and plants. The type II CRISPR effector Cas9 is a RNA-guided endonuclease which recognizes the target DNA sequence by Watson–Crick base pairing with an engineered single-guide RNA (sgRNA), and the protospacer adjacent motifs (PAMs) adjacent to the targets are necessary for target recognition by sgRNA/Cas9 complex (Cong et al. 2013). The commonly used *Streptococcus pyogenes* Cas9 (SpCas9), for example, generally recognizes NGG PAMs, though targets with NAG PAMs were also reported to be cleaved by SpCas9 (Meng et al. 2018). As genome editing technologies have advanced, novel CRISPR effectors such as Cas12a (also known as Cpf1), Cas12f, and CasΦ have been developed for targeted DNA cleavage with different PAMs (Zetsche et al. 2015; Pausch et al. 2020; Wu et al. 2021).

Cas12a belongs to Class 2 type V CRISPR effector and recognizes T-rich PAMs, which are located at 5’ end of the target sites (Zetsche et al. 2015). Unlike Cas9 nuclease, Cas12a has RNase III activity and can process precursor CRISPR RNA (pre-crRNA) to produce mature crRNA, which is much shorter (~ 43 nt) than sgRNA (~ 100 nt) for Cas9 (Zaidi et al. 2017; Zetsche et al. 2017). Cas12a is guided by a single crRNA to search and cleave the target sequences without the need of *trans*-acting crRNA (Zetsche et al. 2015, 2017). Up to now, Cas12a proteins from *Acidaminococcus sp. BV3L6* (AsCpf1/Cas12a), *Francisella novicida* (FnCpf1/Cas12a), and *Lachnospiraceae bacterium ND2006* (LbCpf1/Cas12a) have been used for precise genome editing in multiple plants, including *Arabidopsis*, rice, soybean, tobacco, citrus, and cotton (Endo et al. 2016; Xu et al. 2017; Wang et al. 2017; Kim et al. 2017; Jia et al. 2019; Li et al. 2019).

:The grapevine (*Vitis vinifera* L.) is an economically important fruit crop worldwide, and grape berries are a good source of anthocyanins, resveratrol, and flavonoids, both of which are beneficial to human health (Di Lorenzo et al. 2021). However, improvement of grapevine traits so far largely relies on conventional breeding, which is laborious and time-consuming to develop desired cultivars (Ren et al. 2022). The CRISPR/Cas9-mediated genome editing technology has emerged as an efficient and promising approach for crop improvement (Manghwar et al. 2019; Zhu et al. 2020). As a significant genome editing technique, the CRISPR/Cas9 system has also been widely used for gene functional study and trait improvement in grapevine these years (Ren et al. 2016, 2020, 2021; Wang et al. 2018; Li et al. 2020; Sunitha and Rock 2020; Clemens et al. 2022; Scintilla et al., 2022). However, in some cases Cas9 protein could be less efficient in genome engineering than Cas12a. For instance, a recent study showed that CRISPR/Cas12a exhibits robust activity in destroying *cis*-regulatory elements and therefore represents a promising tool for promoter editing (Zhou et al. 2023). Nevertheless, the efficacy and efficiency of CRISPR/Cas12a in grapevine remains unexplored to date. In this study, we demonstrated that CRISPR/LbCas12a system could induce efficient targeted mutagenesis in grapevine and the editing efficiencies caused by LbCas12a were improved by short-term heat treatment. Furthermore, the crRNAs sequences are important to efficient editing during multiplex genome editing. Moreover, the crRNA length also had an effect on genome editing in grapevine. All these results demonstrate that CRISPR/LbCas12a is an efficient system for targeted genome editing in grapevine and hence would facilitate the application of this editing tool in this important fruit crop in the future.

## Results

### CRISPR/LbCas12a is efficient in generating targeted mutagenesis in grapevine

**Fig. 1:**
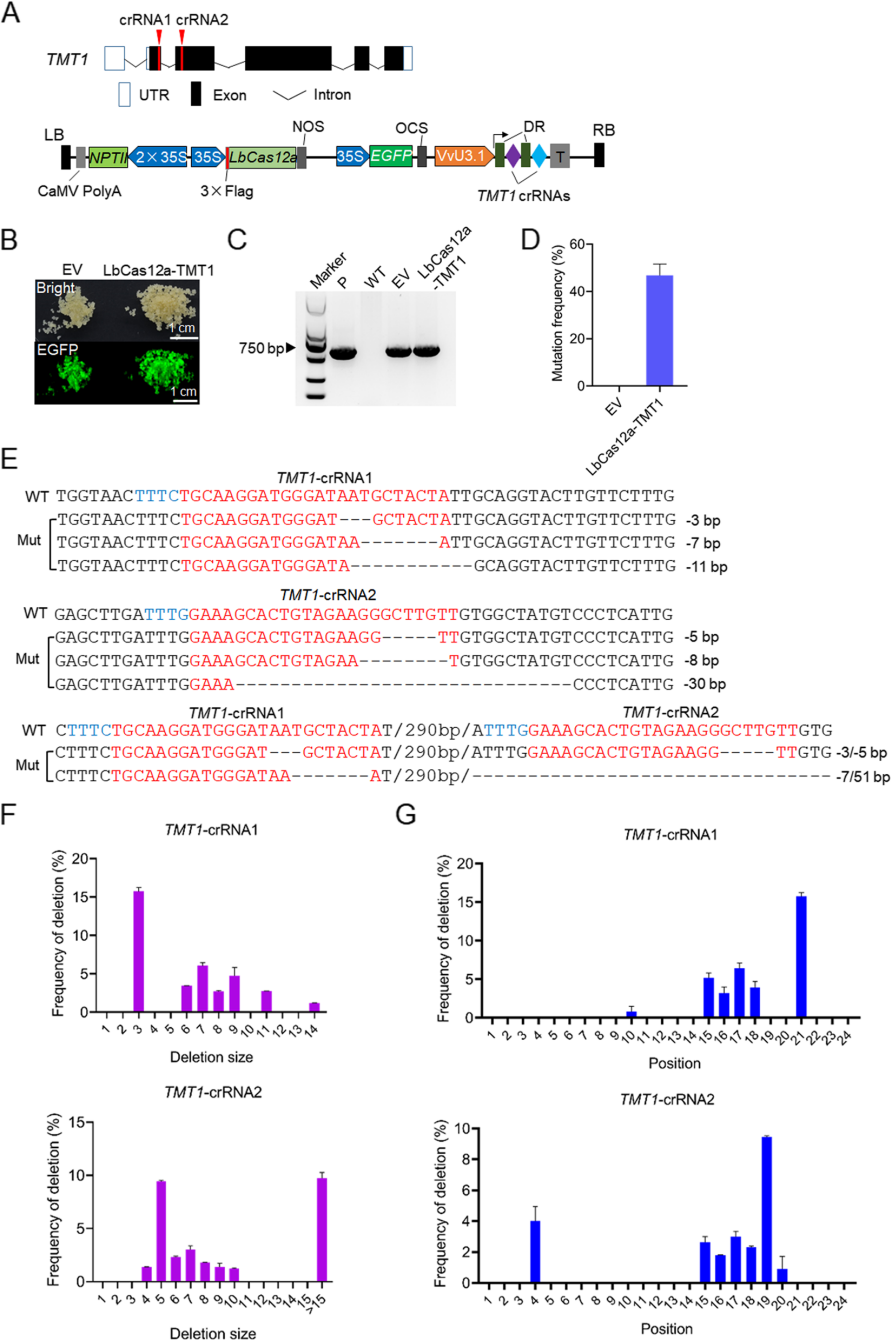
Targeted editing of *TMT1* gene in 41B grape cells. **A** Schematic illustration of target design and LbCas12a expression vector. *NPT*II, neomycin phosphotransferase gene; NOS, terminator of nopaline synthase gene; OCS, terminator of octopine synthase gene; EGFP, enhanced green fluorescent protein; DR, LbCas12a direct repeat; LB, left border; RB, right border. **B** Transformed 41B cells with EGFP fluorescence. The LbCas12a vector containing the *TMT1* crRNA array (LbCas12a-TMT1) and empty vector (EV) without crRNA were introduced into 41B cells, respectively. **C** Identification of exogenous T-DNA insertions by PCR. LbCas12a-specific primers were used to identify the T-DNA in 41B cells. The CRISPR vector and wild-type (WT) cells were used as positive (P) and negative controls, respectively. **D** Mutation efficiencies detected for EV and LbCas12a-TMT1 cells. **E** Representative mutated sequences detected at the two targets in *TMT1* gene. The target sequences and PAMs are indicated in red and blue, respectively. The mutation types are shown on the right. WT, wild-type sequences; Mut, mutated sequences. **F** Deletion size of nucleotides observed at the two targets in *TMT1* gene. **G** Positions of nucleotide deletions happened at the two targets in *TMT1* gene. The first base adjacent to the PAM is referred to as position 1. Data are collected from three replicates and shown as means ± SD

To test the efficacy of CRISPR/LbCas12a system in grapevine, we first amplified the coding sequence of LbCas12a from the LbCas12a-OsU6 vector (Wang et al. 2017) and put it downstream of a CaMV 35S promoter (Fig. 1A). Then we designed two crRNAs targeting the *tonoplastic monosaccharide transporter 1* (*TMT1*) gene, and the two *TMT1* crRNAs interspaced by LbCas12a direct repeat (DR) were ligated together to form a single crRNA array (Fig. 1A). The expression of crRNAs is driven by the grape VvU3.1 promoter (Ren et al. 2021) (Fig. 1A). The LbCas12a expression vector harboring the *TMT1* crRNA array (referred to as LbCas12a-TMT1), as well as the empty vector (EV) without crRNA array, was introduced into 41B embryogenic grape cells via *Agrobacterium*-mediated transformation. The presence of an enhanced green fluorescent protein (EGFP) gene expression cassette in editing vectors enables us to detect the transformed cells according to the EGFP fluorescence (Fig. 1B). Identification of exogenous T-DNA insertions by PCR using *LbCas12a*-specific primers further confirmed the successful transformation of 41B cells (Fig. 1C). To detect the mutations in *TMT1* gene, the fragments containing the designed targets were amplified from transformed cells and analyzed by Hi-TOM assay (Liu et al. 2019). As expected, the two designed targets within the *TMT1* gene in LbCas12a-TMT1 transformed cells were successfully edited with an overall editing efficiency of 46.8%; by contrast, the EV cells were detected with no indel mutations (Fig. 1D, Supplementary Table S1). Interestingly, all the identified mutations are nucleotide deletions, with the deletion size being over 3 bp (Fig. 1E-F, Supplementary Table S1). Large deletions (> 10 bp) were also observed, and around 4% of 51-bp deletions, for example, were detected at the *TMT1*-crRNA2 site (Fig. 1F, Supplementary Table S1). Analysis of the deletion positions revealed that deletions primarily happened at 15 ~ 21nt within the 24 nt target sequences (Fig. 1G, Supplementary Table S1).

**Fig. 2:**
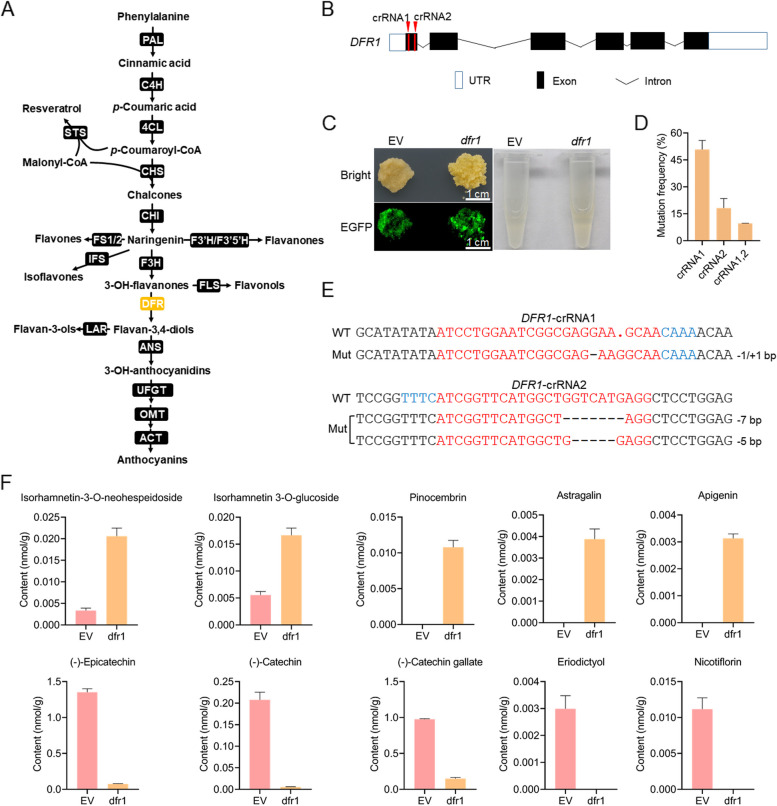
Knockout of *DFR1* gene affects flavonoids accumulation in grape cells. **A** Schematic of the major branch pathways of flavonoid biosynthesis, starting with phenylpropanoid metabolism. The enzymes involved in the pathways are abbreviated as follows: Phe ammonia-lyase (PAL), cinnamate-4-hydroxylase (C4H), 4-coumaroyl:CoA-ligase (4CL), chalconesynthase (CHS), chalcone isomerase (CHI), flavone synthase (FS1 and FS2), flavonoid 3’ hydroxylase (F3’H), flavonoid 3′5’ hydroxylase (F3′5’H), flavanone 3-hydroxylase (F3H), isoflavone synthase (IFS), flavonol synthase (FLS), dihydroflavonol-4-reductas (DFR), stilbene synthase (STS), anthocyanidin synthase (ANS), UDP-glucose flavonoid glucosyltransferase (UFGT), *O*-methyltransferase (OMT), leucoanthocyanidin reductase (LAR), acyltransferases (ACT). **B** Target design for *DFR1* editing. **C** Photos of grape cells and ethanol extracts of EV and *dfr1* mutant cells. **D** Mutation frequencies detected at the two targets in *DFR1* gene. **E** Representative mutated sequences detected at the two targets in *DFR1* gene. The target sequences and PAMs are indicated in red and blue, respectively. The mutation types are shown on the right. **F** Flavonoid compounds with significant changes in contents in *dfr1* cells. Data are shown as means ± SD (n = 3)

In addition to the *TMT1* gene, we also targeted the *dihydroflavonol-4-reductase 1* (*DFR1*) gene, which is involved in biosynthesis of flavonoids through the phenylpropanoid pathway (Fig. 2A). *DFR1*, together with *DFR2*, was highly expressed in 41B cells based on the transcriptome data (Supplementary Table S2). However, difference was observed in coding sequence of *DFR2* in different grape genome annotations (Supplementary Fig. S1), and amplification of *DFR2* gene from 41B cells was failed by PCR (data not shown). Hence, the *DFR1* was chosen as the target and two crRNAs were designed to target the first exon of *DFR1* gene (Fig. 2B). *dfr1* mutant cells were successfully obtained after transformation (Fig. 2C-E, Supplementary Table S3). The editing efficiencies detected for *DFR1*-crRNA1 and *DFR1*-crRNA2 were 50.8% and 18.2%, respectively (Fig. 2D). Interestingly, the predominant mutation type detected at *DFR1*-crRNA1 was 1-bp indels, whereas the mutations detected at *DFR1*-crRNA2 were ≥ 3-bp deletions (Fig. 2E, Supplementary Table S3). Difference was observed in the color of grape cells and ethanol extracts of EV and *dfr1* cells (Fig. 2C), which suggested that the accumulation of flavonoids in *dfr1* cells might have been changed. To investigate the changes in flavonoids accumulation in *dfr1* cells, we conducted targeted metabolomics to determine the contents of a total of 185 flavonoid compounds in the grape cells (Supplementary Table S4). Analysis of differentially accumulated compounds revealed that 15 compounds were significantly up- or down-regulated in *dfr1* cells (Supplementary Table S5). Among them, isorhamnetin 3-O-glucoside (flavonol), isorhamnetin-3-O-neohespeidoside (flavonol), astragalin (flavonol), apigenin (flavone), and pinocembrin (flavanone) were obviously accumulated in *dfr1* cells (Fig. 2F, Supplementary Table S5). By contrast, the downstream flavanols such as (-)-catechin gallate, (-)-catechin, and (-)-epicatechin were significantly decreased in *dfr1* cells (Fig. 2F, Supplementary Table S5).

Taken together, the successful editing of *TMT1* and *DFR1* genes in 41B grape cells demonstrated that CRISPR/LbCas12a is efficient in inducing targeted mutagenesis in grapevine.

### CRISPR/LbCas12a-mediated genome editing could be improved by short-term heat treatment

**Fig. 3:**
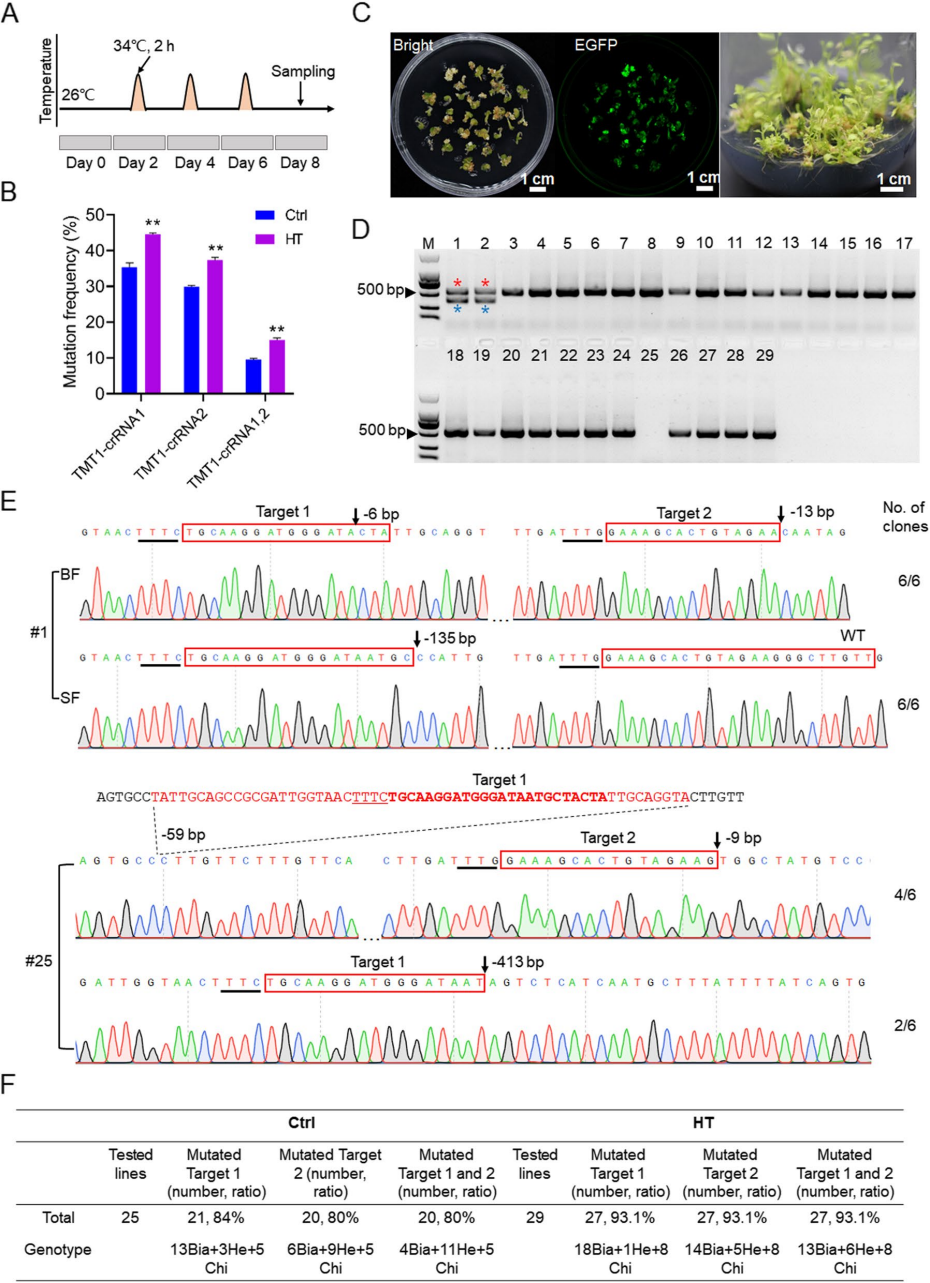
Heat treatment improves editing efficiency using LbCas12a in grapevine. **A** Schematic diagram of heat treatment. **B** Mutation frequencies at the two targets in *TMT1* gene under control (Ctrl, 26℃) and heat treatment (HT, 34℃) conditions. ** indicates a highly significant (*P* < 0.01) difference determined by Student’s *t*-test. **C** Regeneration of grapevine plants. Plant regeneration from 41B cells after HT was shown as example. Embryos developed from transformed grape cells were detected with EGFP fluorescence. Whole plants were recovered from germinated embryos on regeneration medium. **D** Amplification of target fragments from transgenic grapevine plants. The PCR results obtained with HT plants were shown. Lanes 1–29 represent samples from 29 independent grapevine plants. The desired bands (referred to as big fragment, BF) are indicated in red asterisks, and smaller fragments (SF, indicated in blue asterisks) were also observed in lane 1 and 2. M, DNA marker. **E** Sanger sequencing results of the target fragments in Plant #1 and #25. Representative chromatograms of mutated sequences are shown. The target sequences are indicated in red boxes, and the PAMs are underlined. The deletion positions are denoted by black arrows, and mutation types are shown behind the arrows. The numbers of amplicons identified from 6 analyzed clones are shown on the right. The missing sequence identified in plant #25 is indicated in red, in which the target sequence is indicated in bold. **F** Overview of targeted mutagenesis under Ctrl and HT conditions. Bia, biallelic mutants; He, heterozygotes; Chi, chimeras

Previous studies have revealed that the activity of LbCas12a was affected by temperatures (Kleinstiver et al. 2019; Li et al. 2021). To test the effect of temperatures on CRISPR/LbCas12a-mediated genome editing in grapevine, the 41B cells transformed with LbCas12a-TMT1 were treated at 34℃ for 2 h once per day for a total of 3 days during a 7-day culture (Fig. 3A), while the cells cultured at 26℃ were sampled as the control. Editing efficiencies were then analyzed to evaluate the effect of heat treatment (HT) on genome editing. Compared with the control, HT obviously improved the editing efficiencies at *TMT1*-crRNA1 and *TMT1*-crRNA2 sites, with the editing efficiencies of *TMT1*-crRNA1 and *TMT1*-crRNA2 increasing from 35.3% to 44.6% and 29.9% to 37.3%, respectively (Fig. 3B, Supplementary Table S6). Notably, the percentage of mutated sequences containing simultaneous mutations at *TMT1*-crRNA1 and *TMT1*-crRNA2 was also significantly increased from 9.6% to 15.1% after HT (Fig. 3B, Supplementary Table S6).

The grape cells after HT, together with the cells under control (Ctrl) conditions, were used for regeneration to develop the whole grapevine plants (Fig. 3C). A total of 29 and 25 plants were identified as transgenic plants by PCR for HT and Ctrl, respectively (Supplementary Fig. S2). The target fragments of *TMT1* gene were amplified using the primers TMT1-F1/R1 (Supplementary Fig. S3) from these transgenic plants and analyzed by Sanger sequencing. Intriguingly, the desired PCR bands in most plants were specific, whereas smaller bands were also observed in two lines, namely line #1 and #2 (Fig. 3D). The generation of bands with different sizes in line #1 and #2, as well as the lack of desired product in line #25, for instance, might be explained by large deletions in these plants, which result in the reduction in PCR product size or disruption of primers binding sites. Sequencing results showed that there are two types of mutations at the designed targets in *TMT1* gene in line #1, one of which is 135-bp deletion at the first target (Target 1). In addition, 6- and 13-bp deletions were also identified at Target 1 and Target 2 simultaneously (Fig. 3E). For line #25, we redesigned the primers (TMT1-F2/R2) for amplification of a 1136-bp target region of *TMT1* gene (Supplementary Fig. S3), and 59- and 413-bp deletions were detected at Target 1 (Fig. 3E). These sequencing results are compatible with the PCR results (Fig. 3D). Analysis of mutations in regenerated grapevine plants showed that HT improved the genome editing by LbCas12a, and the editing efficiencies at Target 1/Target 2 and both of the two sites were all increased (Fig. 3F). Importantly, the percentage of edited plants with biallelic mutations at Target 1 and 2 was increased from 20 to 48% after HT (Fig. 3F). All these results suggested that short-term HT could be a promising approach to improve editing efficiencies when using LbCas12a in grapevine.

### The target sequence is the primary factor affecting editing efficiency during multiplex genome editing

**Fig. 4:**
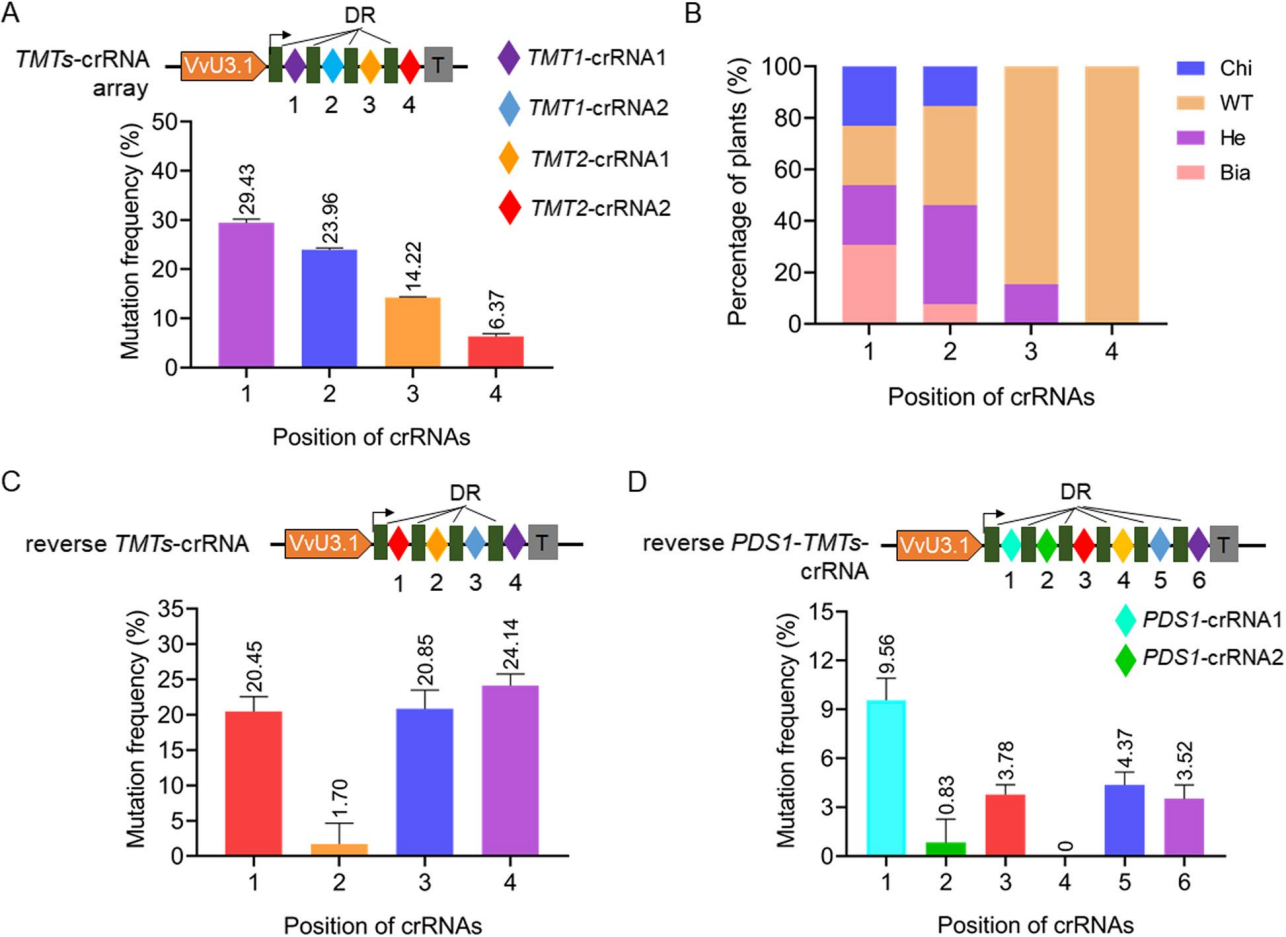
Evaluation of the effect of crRNA positions on editing efficiencies during multiplex genome editing. **A** Mutation frequencies mediated by LbCas12a in 41B cells using the *TMTs*-crRNA array. Bars are mean of 3 technical replicates from one experiment. Error bars are SD. DR, direct repeat. **B** Percentage of edited plants regenerated from 41B cells in A. Chi, chimera; WT, wild type; He, heterozygotes; Bia, biallele mutants. **C** Mutation frequencies mediated by LbCas12a in 41B cells using the reverse *TMTs*-crRNA array. The positions of crRNAs in the array were altered by exchanging the order of crRNAs. **D** Mutation frequencies mediated by LbCas12a in 41B cells using the reverse *PDS1*-*TMTs*-crRNA array. Data are shown as means ± SD from three technical replicates

During the editing of *TMT1* gene, we found that the editing efficiencies for *TMT1*-crRNA1 were always higher than that for *TMT1*-crRNA2 before and after HT in grape cells (Fig. 3B). Accordingly, the ratios of biallelic mutations for *TMT1*-crRNA1 were also higher than that observed for *TMT1*-crRNA2 in transgenic grapevine plants (Fig. 3F). We therefore speculated that the editing efficiency might be affected by the difference of target sequences and/or the positions of crRNAs within the crRNA array. To test this hypothesis, we designed two crRNAs targeting the *TMT2* gene and combined them with previously developed *TMT1*-crRNAs to make the *TMTs*-crRNA array (Fig. 4A, Supplementary Fig. S4). The resulting editing vector was introduced into 41B cells, and targeted mutagenesis was successfully detected at the four targets (Supplementary Fig. S4, Table S7). Simultaneous knockout of *TMT1* and *TMT2* resulted in reduced sugar accumulation in grape cells (Supplementary Fig. S4), which is consistent with the previous results obtained using CRISPR/Cas9 (Ren et al. 2021). The editing efficiencies with respect to the four individual targets were evaluated, and the results showed that the highest editing efficiency was achieved by using *TMT1*-crRNA1 (29.43%), which is placed at position 1 (the most promoter-proximal crRNA) in the *TMTs*-crRNA array (Fig. 4A, Supplementary Table S7). On the contrary, the *TMT2*-crRNA2 at position 4 resulted in the lowest editing efficiency (6.37%) (Fig. 4A, Supplementary Table S7). Furthermore, analysis of 13 transgenic grapevine plants showed that the highest percentage of edited plants, including biallele mutants, heterozygotes, and chimeras, was observed with *TMT1*-crRNA1, whereas no mutated plants were detected for *TMT2*-crRNA2 (Fig. 4B, Supplementary Fig. S5). To further confirm our hypothesis, we exchanged the order of crRNAs to make the reverse *TMTs*-crRNA array without changing the target sequences (Fig. 4C). If the editing is just influenced by the order of crRNAs, the editing profiles of the crRNAs at the same positions within the array are expected to be unchanged or less affected irrespective of the target sequences. However, the results obtained using the reverse *TMTs*-crRNA array showed that the two crRNAs designed for *TMT1* still resulted in higher mutation frequencies (24.14% for *TMT1*-crRNA1 at position 4 and 20.85% for *TMT1*-crRNA2 at position 3) when compared with the two *TMT2*-crRNAs (20.45% for *TMT2*-crRNA2 at position 1 and 1.7% for *TMT2*-crRNA1 at position 2) (Fig. 4C, Supplementary Table S8). In addition, two *phytoene desaturase 1* (*PDS1*) crRNAs were designed and added to the reverse *TMTs*-crRNA array to develop the reverse *PDS1*-*TMTs*-crRNA array (Fig. 4D, Supplementary Fig. S6). The newly developed crRNA array was introduced into 41B cells with LbCas12a and editing efficiencies were evaluated by using Hi-TOM assay. The editing profiles resulted by the four *TMTs* crRNAs in the reverse *PDS1*-*TMTs*-crRNA array was similar to that using the reverse *TMTs*-crRNA array as shown in Fig. 4C, though the positions of the four crRNAs had been altered (Fig. 4D, Supplementary Table S9). All these results indicated that the target sequence is the predominant factor affecting editing efficiencies during multiplex genome editing.

### The crRNA length has an effect on LbCas12a-mediated genome editing in grape

**Fig. 5:**
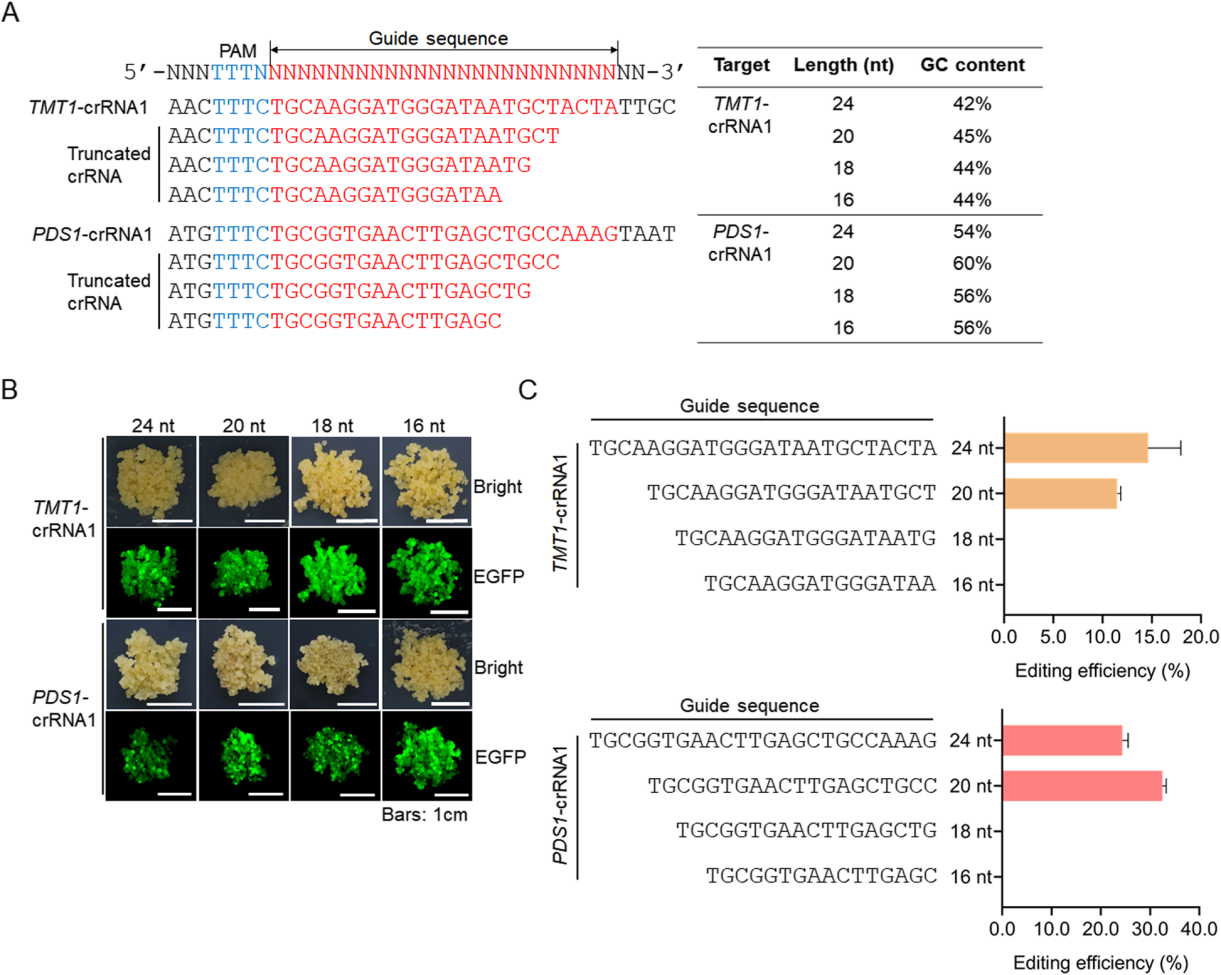
Assay of the activities of truncated crRNAs (trucrRNAs) in inducing targeted mutagenesis. **A** Design of trucrRNAs. The GC contents of trucrRNAs are shown on the right. **B** Transformed grape cells with EGFP fluorescence. **C** Editing efficiencies using trucrRNAs with different guide length. Data are shown as means ± SD from three technical replicates

The crRNA guide sequences designed using online tools such as CRISPR-GE (Xie et al. 2017) are generally 24 nt in length. To test whether a truncated crRNA (trucrRNA) could guide LbCas12a to cleave the target sequence, we developed 3’-truncated 16, 18, and 20 nt guides based on the initially designed 24 nt *TMT1*-crRNA1 and *PDS1*-crRNA1, respectively (Fig. 5A). The decrease in crRNA guide length has little impact on GC content (Fig. 5A). These truncated crRNAs as well as the original crRNAs were introduced into 41B cells, respectively, with LbCas12a and transformed cells were recovered according to the EGFP fluorescence (Fig. 5B). The editing of the targets in these cells was detected by using Hi-TOM assay. The results showed that 20 nt guides of trucrRNAs for *TMT1* and *PDS1* could efficiently induce targeted mutagenesis at the targets, and the resulting efficiencies were comparable with that using crRNAs with 24 nt guide sequences (Fig. 5C, Supplementary Table S10). However, for 16 and 18 nt guide trucrRNAs, no detectable mutations were observed at the designed targets (Fig. 5C, Supplementary Table S10). It has been reported that truncated sgRNAs could improve the specificity of Cas9 by reducing off-target effects (Fu et al. 2014). Here we predicted the putative off-target sites for the original and truncated crRNAs based on sequence similarity (Supplementary Table S11), and seven predicted off-target sites were randomly selected and analyzed by Hi-TOM assay. According to the results, no indel mutations were observed at these predicted off-target sites (Supplementary Table S12). These results revealed that LbCas12a-mediated genome editing is specific in grapevine, and trucrRNAs with 20 nt guide sequences are efficient in inducing targeted genome editing, while trucrRNAs with shorter regions of target complementarity ≤ 18 nt in length may not induce mutagenesis at desired targets.

## Discussion

### CRSIRP/LbCas12a system primarily induces nucleotide deletions in grapevine

Though three Cas12a proteins, namely *Acidaminococcus* sp. BV3L6 Cas12a (AsCas12a), *Francisella novicidain U112* Cas12a (FnCas12a), and LbCas12a, have been applied in plants, LbCas12a outperforms the others with better overall editing efficiencies (Endo et al. 2016; Xu et al. 2017; Tang et al. 2017; Zhong et al. 2018). In the present study, we demonstrated the efficacy of CRSIRP/LbCas12a in genome editing in grapevine, and interestingly, almost all the mutations induced by LbCas12a are nucleotide deletions, with most of deletions being ≥ 3 bp (Figs. 1E, 2E and 3E, Supplementary Table S1, S3). The type of mutations observed with LbCas12a in grapevine is quite different from that using SpCas9, which typically resulted in 1-bp insertions and short nucleotide (≤ 3 bp) deletions (Ren et al. 2016). Moreover, LbCas12a-induced mutations primarily happened at 15–21 nt downstream of the PAMs (Fig. 1G), which may be associated with the staggered cutting of LbCas12a at 18–23 bases within the protospacers (Zetsche et al. 2015).

### The target sequences have an effect on editing efficiency

Editing efficiencies at different targets ranged from 6.4% (*TMT2*-crRNA2) to 50.8% (*DFR1*-crRNA1) in grape cells (Figs. 2D and 3A). In grapevine plants, LbCas12a-mediated mutation frequencies were over 80%, and HT could improve editing efficiencies (Fig. 3F). Recently, the temperature-tolerant LbCas12a (ttLbCas12a) has been developed with enhanced editing activity (Schindele and Puchta 2020), which could serve as an alternative for genome editing in grapevine. Genome editing with Cas9 revealed that the efficiencies might be affected by target sequences (Ren et al. 2016). In the current study, the two *TMT1* crRNAs exhibited relatively higher mutation efficiencies during the editing (Fig. 4), probably due to the high activities of the two crRNAs. The changes in target sequence could alter RNA secondary structure, which is a fundamental parameter that affects the activity of CRISPR systems (Kocak et al. 2019). We analyzed the secondary structures of these designed crRNAs and found that the two *TMT1* crRNAs, particularly the *TMT1* crRNA1, have low possibilities to form stable secondary structures (Supplementary Fig. S7), which might account for the high activities of the two crRNAs. Interestingly, the editing efficiency of *TMT2* crRNA2, which resulted in the lowest editing efficiencies in both grape cells and plants in the *TMTs*-crRNA array, was dramatically improved when placed at position 1 in the reverse *TMTs*-crRNA array (Fig. 4A, C); moreover, the *PDS1* crRNA1, which is predicted to have a stable RNA secondary structure (Supplementary Fig. S7), also resulted in the highest editing efficiency in the reverse *PDS1*-*TMTs*-crRNA array (Fig. 4D). Previous study showed that crRNA at position 1 exhibited the highest transcript abundance after in vivo processing of AsCpf1 pre-crRNA transcript in mammalian cells (Zetsche et al. 2017). These results suggest that the editing efficiencies might also be associated with the transcript abundance of mature crRNAs.

### crRNAs with over 18 nt guide sequences are effective in inducing targeted mutagenesis in grape

For LbCas12a, both pre-crRNAs and mature crRNAs were successfully used for genome editing in rice (Xu et al. 2017; Wang et al. 2017). A mature crRNA for LbCas12a consists of a 20 nt DR and a designed guide sequence (Hu et al. 2017; Kleinstiver et al. 2019). In mammalian cells, crRNAs with 23–25 nt guide sequences were used for genome editing (Zetsche et al. 2015). Similarly, in plants, 22–25 nt guide sequences were generally designed for targeted mutagenesis (Xu et al. 2017; Wang et al. 2017; Tang et al. 2017; Hsu et al. 2019; An et al. 2020; Duan et al. 2021; Li et al. 2021). However, previous study showed that 4–6 bases at 3’ end of the crRNA guide sequence are insensitive to base mismatches (Kleinstiver et al. 2016), suggesting that these bases may be not necessary for efficient DNA cleavage. Here we tested the activities of trucrRNAs of *TMT1* crRNA1 and *PDS1* crRNA1 with 16, 18, and 20 nt guide sequences in inducing targeted mutagenesis in grape cells and found that trucrRNAs with 20 nt guide were efficient in inducing mutations, whereas crRNA targets ≤ 18 nt cannot result in detectable mutations at target sites (Fig. 5C, Supplementary Table S10). These results are compatible with previous finding that positions 21–24 (with 1 being the first base adjacent to the PAM) do not participate in the formation of crRNA-DNA heteroduplex (Yamano et al. 2016). Additional in vivo experiments should be conducted in the future to evaluate the relationship between crRNA length and editing efficiency. For Cas9, truncated sgRNAs abolish the cleavage of DNA sequences without affecting the binding of Cas9 protein to the targets, which allows for the development of versatile CRISPR/Cas9 system for transcriptional regulation by using truncated sgRNAs (Pan et al. 2022). Whether this mechanism is applicable to CRISPR/LbCas12a is still unknown and should be further confirmed in the future.

### CRSIRP/LbCas12a system has great potential in engineering metabolic pathway

As the earliest developed CRISPR/Cas system, CRISPR/Cas9 has been used for application of metabolic engineering in plants, such as manipulation of carotenoid and γ-aminobutyric acid metabolic pathways (Li et al. 2018a, 2018b). In this study, simultaneously mutating *TMT1* and *TMT2* genes using LbCas12a resulted in reduced sugar accumulation in grape cells (Supplementary Fig. S4). Furthermore, the *DFR1* gene involved in biosynthesis of flavonoids, which has drawn great attention due to their extensive biological applications (Wen et al. 2021), was also knocked out by using CRISPR/LbCas12a. The phenylpropanoid pathway is associated with biosynthesis of resveratrol, flavonoids, and anthocyanins in grapevine (Fig. 2A). Analysis of expression profiles of genes encoding key enzymes in the pathway showed that the expression of *stilbene synthase genes* (*STSs*) and *UDP-glucose flavonoid glycosyltransferases* (*UFGT*) could hardly detected in 41B cells (Supplementary Table S2), suggesting that biosynthesis of resveratrol and anthocyanins is blocked in 41B cells. We therefore speculated that knockout of the *DFR1* gene may retard the biosynthesis of downstream flavanols and promote the accumulation of upstream flavonoids in 41B cells (Fig. 2A). The results of metabolomics showed that multiple compounds belonging to flavonols, flavanones, and flavones, for example, were increased in *dfr1* cells, whereas several flavanol compounds were found to be decreased (Supplementary Fig. S8). Among them, 15 compounds such as isorhamnetin 3-O-glucoside, isorhamnetin-3-O-neohespeidoside, and astragalin were differentially enriched in *dfr1* cells (Fig. 2F, Supplementary Table S5). The results obtained here provide a promising way to produce valuable compounds by modifying metabolic pathways using CRISPR/LbCas12a in suspension-cultured grape cells, which have been extensively used to synthesize a variety of secondary metabolites (Pietrowska-Borek et al. 2021; Badim et al. 2022). Given that both sugars and flavonoids contribute greatly to grape berry quality, the results of this study suggest that CRISPR/LbCas12a could be an important tool for trait improvement in grapevine.

## Conclusion

Our results obtained in this study demonstrate that CRISPR/LbCas12a is a powerful genome editing tool with promising applications in grapevine, and the use of this CRISPR technique is expected to facilitate the basic and applied research in this important fruit crop.

## Materials and methods

### Plant material, transformation and regeneration

The 41B (*V. vinifera* × *V. berlandieri*) embryogenic suspension cells were used in this study. The grape cells were sub-cultured weekly in glycerol-maltose (GM) liquid medium containing 1 mg/L naphthoxy acetic acid (NOA). For transformation of grape cells, 1 mL of the 41B suspension cells were incubated with *Agrobacterium* cells harboring the desired plasmids at 28℃ for 30 min, then the grape cells were cultured on solid GM in the dark for 2 days. After transformation, the grape cells were cultured in liquid GM supplemented with 200 mg/L timentin and 5 mg/L paromomycin for at least 2 months for selection of transformed cells. For plant regeneration, antibiotic-resistant cells were transferred to regeneration medium (GM without NOA) for induction of embryos. The induced embryos further germinated on regeneration medium and whole plants were recovered with roots in McCown woody plant medium (Duchefa Biochemie) supplemented with 0.2 mg/L naphthylacetic acid (NAA). Transformation of grape cells and plant regeneration were performed as previously described (Osakabe et al. 2018; Ren et al. 2020).

### Plasmid construction

To develop the LbCas12a expression vector, the coding sequence of LbCas12a was amplified from LbCas12a-OsU6 construct (Wang et al. 2017) by PCR using the primers LbCas12a-PCR-F/R (Supplementary Table S13). The amplified sequence was cloned into the modified pCAMBIA2300-EGFP vector, in which the *LbCas12a* gene was driven by a 35S promoter, through *Eco*RI and *Kpn*I sites via homologous recombination (HR) by using the ClonExpress II One Step Cloning Kit (Vazyme). The constructed vector was named pCAMBIA2300-LbCas12a. To design the guide sequences, the exons of *PDS1* (VIT_09s0002g00100), *TMT1* (VIT_18s0122g00850), *TMT2* (VIT_03s0038g03940), and *DFR1* (VIT_18s0001g12800) were used as input sequences for target design, respectively, using the CRISPR-GE online tool (http://skl.scau.edu.cn/) (Xie et al. 2017). Designed targets with DR sequences (Supplementary Fig. S9-S10) were commercially synthesized (Tsingke) and ligated into the pCAMBIA2300-LbCas12a vector under the control of the VvU3.1 promoter (Ren et al. 2021) through *Hin*dIII site using the T4 DNA ligase kit (NEB). The trucrRNAs expression cassettes were developed according to the previous study (Ren et al. 2016). In brief, a VvU3.1-DR-HDV (hepatitis delta virus ribozyme) fragment was first synthesized (Tsingke) and inserted into the pCAMBIA2300-LbCas12a vectorvia *Hin*dIII site. Then the truncated guide sequences were synthesized as reverse primers (Supplementary Table S13) and fused to the VvU3.1 promoter by PCR amplification. The amplified VvU3.1-DR-guide fragments were finally cloned into the *Eco*RI- and *Hin*dIII-digested pCAMBIA2300-LbCas12a vector through HR in place of the VvU3.1 promoter. The developed plasmids were introduced into *Agrobacterium* strain EHA105 for grape cells transformation.

### Identification of transformants and heat treatment

To identify transformed grape cells, the cells were collected from liquid medium and detected with a CCD camera (Tanon 5200) to check the EGFP fluorescence. Then gDNA was isolated from grape cells and used as template for PCR identification with *LbCas12a*-specific primers (Supplementary Table S13). For regenerated grapevine plants, leaves were sampled for gDNA preparation, and transgenic plants were identified by PCR using *LbCas12a*-specific primers as well. For heat treatment, the grape cells from one flask were evenly transferred to two 100 mL flasks fitted with 30 ml of fresh GM medium (day 0) and shaken at 120 rpm at 26℃ in the dark for seven days (day 7). During the 7-d culture, one of the two flasks was transferred to a shaker set to 34℃ and incubated for 2 h on day 1, 3, and 5, respectively. The other was kept at 26℃ as the control. The treated and control cells were harvested on day 7 for gDNA isolation. The treatment was conducted in three replicates.

### Mutation detection

To detect the mutations at the designed targets, the sequences containing the target sites were first amplified by PCR using site-specific primers from grape cells or grapevine plants. Mutations were then detected using Sanger sequencing or high-throughput sequencing. The mutations in grapevine plants were detected using Sanger sequencing. For Sanger sequencing assay, the amplified target fragments were ligated into pLB vector (TIANGEN), and a number of 6 clones were randomly picked and analyzed by Sanger sequencing. The mutations in 41B cells were detected using Hi-TOM assay (http://www.hi-tom.net/hi-tom/). For Hi-TOM assay, target-specific primers with bridging sequences at 5’ end were used to amplify the target fragments using the rapid Taq master mix (Vazyme) according to the manufacturer’s protocol. The PCR products were used for NGS after a second-round PCR called barcoding PCR. The mutations were determined by analyzing the sequencing reads according to the protocol described by Liu et al., (2019). All the primers are available in Supplementary Table S13.

### Determination of flavonoids and sugars

Grape cells were freeze-dried and ground into powder with liquid nitrogen. An amount of 20 mg of powder was used for extraction with 0.5 mL of 70% methanol. After that, 10 μL of standard compounds (4,000 nmol/L) were added into the extract as internal standards for quantification. The extract was sonicated for 30 min and centrifuged at 12,000 × *g* at 4℃ for 5 min. The supernatant was filtered through a 0.22 μm membrane filter before liquid chromatography tandem mass spectrometry (LC–MS/MS) analysis. Flavonoids were detected with Applied Biosystems Sciex QTRAP 6500 LC–MS/MS platform in Wuhan MetWare Biotechnology Co., Ltd. Hierarchical cluster analysis of samples and metabolites were conducted and visualized as heatmap using the *R* package pheatmap. To identify differentially enriched metabolites, the data was log-transformed (log2) and mean centering, followed by orthogonal partial least squares-discriminant analysis (OPLS-DA), and variable importance in projection (VIP) values were obtained according to the OPLS-DA results using the *R* package MetaboAnalystR. Differentially enriched metabolites between groups were selected based on the VIP values (VIP > 1) and fold changes (FC, FC ≥ 2 or FC ≤ 0.5). Soluble sugars levels were measured using a high performance liquid chromatography (HPLC) system as previously described by Zhang et al. (2019).

## Supplementary Information

**Additional file 1:** **Supplementary Fig. S1.** Sequence alignment of the coding sequence of the *DFR2* gene in different genome annotations. **Supplementary Fig. S2. **PCR identification of transgenic plants regenerated from 41B embryogenic grape cells transformed with LbCas12a-TMT1 without or with heat treatment. **Supplementary Fig. S3.** Sequencing results of* TMT1* editing in LbCas12a-TMT1 grapevine plants. **Supplementary Fig. S4. **Knockout of *TMT1* and *TMT2* genes in grape. **Supplementary Fig. S5.** Sequencing results of* TMT1* and *TMT2 *editing in LbCas12a-TMTs plants. **Supplementary Fig. S6.** Schematic illustration of the targets design for *PDS1*.**Supplementary Fig. S7.** Predication of the secondary structures of *PDS1* and *TMTs* crRNAs. **Supplementary Fig. S8.** Heatmap of the determined compounds in *dfr1* and EV cells. **Supplementary Fig. S9.** Sequences of *TMT1*, *TMTs *(*TMT1* and *TMT2*), reverse *TMTs* and reverse *PDS1-TMTs *crRNAs expression cassettes. **Supplementary Fig. S10.** Sequence of *DFR1* crRNAs expression cassette. **Supplementary Table S1.** Hi-Tom assay of *TMT1* editing results in EV and LbCas12a-TMT1 cells. **Supplementary Table S2.** The expression of *DFRs*, *STSs* and *UFGT* in 41B cells. **Supplementary Table S3. **Hi-Tom assay of the editing results of *DFR1* gene in 41B cells. **Supplementary Table S4. **Flavonoids contents in* dfr1* and EV cells. **Supplementary Table S5.** Differentially regulated flavonoids in *dfr1* cells versus EV cells. **Supplementary Table S6. **Hi-Tom assay of editing efficiencies at *TMT1* targets after heat treatment. **Supplementary Table S7.** Hi-Tom assay of the *TMT1* and *TMT2* targets by using LbCas12a. **Supplementary Table S8. **Analysis of targeted mutagenesis using the reverse *TMTs*-crRNA array. **Supplementary Table S9.** Analysis of targeted mutagenesis using reverse *PDS1*-*TMTs*-crRNA array. **Supplementary Table S10.** Hi-Tom assay of *TMT1* and *PDS1* editing using crRNAs with different length. **Supplementary Table S11.** The putative off-target sites predicted for truncated *TMT1* and *PDS1 *crRNAs. **Supplementary Table S12. **Analysis of genome editing at putative off-target sites. **Supplementary Table S13.** Primers used in this study.

## Data Availability

All relevant data and vectors that support the findings of this study are available from the corresponding author, upon request.

## Abbreviations

CRISPR: Clustered regularly interspaced short palindromic repeats
LbCas12a: *Lachnospiraceae bacterium ND2006* Cas12a protein
TMT1: Tonoplastic monosaccharide transporter1
HT: Heat treatment
crRNA: CRISPR RNA
trucrRNA: Truncated crRNA
DFR1: Dihydroflavonol-4-reductase 1
PAM: Protospacer adjacent motif
PDS1: Phytoene desaturase 1
STS: Stilbene synthase gene
UFGT: UDP-glucose flavonoid glycosyltransferases
