# Correction: Efficient genome editing in grapevine using CRISPR/LbCas12a system

**DOI:** 10.1186/s43897-024-00133-z

**Published:** 2024-12-13

**Authors:** Chong Ren, Elias Kirabi Gathunga, Xue Li, Huayang Li, Junhua Kong, Zhanwu Dai, Zhenchang Liang

**Affiliations:** 1 State Key Laboratory of Plant Diversity and Specialty Crops, Beijing Key Laboratory of Grape Sciences and Enology, Institute of Botany, the Chinese Academy of Sciences, Beijing, 100093 PR China; 2 China National Botanical Garden, Beijing, 100093 PR China; 3 University of Chinese Academy of Sciences, Beijing, 100049 PR China

**Correction** **:** **Mol Horticulture 3, 21 (2023)**

**https://doi.org/10.1186/s43897-023-00069-w**

Following publication of the original article (Ren et al. 2023), the authors reported an error in authors’ affiliations. The full list of affiliations should be corrected from:

Chong Ren^1,2,3^, Elias Kirabi Gathunga^1,2,4^, Xue Li^1,2,4^, Huayang Li^1,2,4^, Junhua Kong^1,2,3^, Zhanwu Dai^1,2,3^ and Zhenchang Liang^1,2,3,5*^

1 Beijing Key Laboratory of Grape Sciences and Enology, Beijing 100093, PR China

2 State Key Laboratory of Plant Diversity and Specialty Crops, Beijing 100093, PR China

3 China National Botanical Garden, Beijing 100093, PR China

4 University of Chinese Academy of Sciences, Beijing 100049, PR China

5 Institute of Botany, the Chinese Academy of Sciences, Haidian District, Nanxin Village 20, XiangshanBeijing 100093, China

To:

Chong Ren^1,2,3^, Elias Kirabi Gathunga^1,2,3^, Xue Li^1,2,3^, Huayang Li^1,2,3^, Junhua Kong^1,2,3^, Zhanwu Dai^1,2,3^, and Zhenchang Liang^1,2,3*^

1 State Key Laboratory of Plant Diversity and Specialty Crops, Beijing Key Laboratory of Grape Sciences and Enology, Institute of Botany, the Chinese Academy of Sciences, Beijing 100093, PR China

2 China National Botanical Garden, Beijing 100093, PR China

3 University of Chinese Academy of Sciences, Beijing 100049, PR China

The original article (Ren et al. 2023) has been updated.
