# Supplementary material for: Efficient genome editing in grapevine using CRISPR/LbCas12a system
Source: Mol Hortic. 2023 Oct 18;3:21. doi: 10.1186/s43897-023-00069-w (PMC10583370; doi:10.1186/s43897-023-00069-w)
Supplement: Supplementary file 1 — Additional file 1: Supplementary Fig. S1. Sequence alignment of the coding sequence of the DFR2 gene in different genome annotations. Supplementary Fig. S2. PCR identification of transgenic plants regenerated from 41B embryogenic grape cells transformed with LbCas12a-TMT1 without or with heat treatment. Supplementary Fig. S3. Sequencing results of TMT1 editing in LbCas12a-TMT1 grapevine plants. Supplementary Fig. S4. Knockout of TMT1 and TMT2 genes in grape. Supplementary Fig. S5. Sequencing results of TMT1 and TMT2 editing in LbCas12a-TMTs plants. Supplementary Fig. S6. Schematic illustration of the targets design for PDS1.Supplementary Fig. S7. Predication of the secondary structures of PDS1 and TMTs crRNAs. Supplementary Fig. S8. Heatmap of the determined compounds in dfr1 and EV cells. Supplementary Fig. S9. Sequences of TMT1, TMTs (TMT1 and TMT2), reverse TMTs and reverse PDS1-TMTs crRNAs expression cassettes. Supplementary Fig. S10. Sequence of DFR1 crRNAs expression cassette. Supplementary Table S1. Hi-Tom assay of TMT1 editing results in EV and LbCas12a-TMT1 cells. Supplementary Table S2. The expression of DFRs, STSs and UFGT in 41B cells. Supplementary Table S3. Hi-Tom assay of the editing results of DFR1 gene in 41B cells. Supplementary Table S4. Flavonoids contents in dfr1 and EV cells. Supplementary Table S5. Differentially regulated flavonoids in dfr1 cells versus EV cells. Supplementary Table S6. Hi-Tom assay of editing efficiencies at TMT1 targets after heat treatment. Supplementary Table S7. Hi-Tom assay of the TMT1 and TMT2 targets by using LbCas12a. Supplementary Table S8. Analysis of targeted mutagenesis using the reverse TMTs-crRNA array. Supplementary Table S9. Analysis of targeted mutagenesis using reverse PDS1-TMTs-crRNA array. Supplementary Table S10. Hi-Tom assay of TMT1 and PDS1 editing using crRNAs with different length. Supplementary Table S11. The putative off-target sites predicted for truncated TMT1 and PDS1 crRNAs. Supplementary Table [file 43897_2023_69_MOESM1_ESM.pdf]

```
DFR2(V2).SEQ   ................................................................................    0
DFR2(V4).SEQ   GCTCTTTCAACTGGTGCTCCATCTCAAAGGCCCACTTACCCATCCTTTGAGACTAAGTTGTGTGAAGTACAGCTTGTTTG  880

DFR2(V2).SEQ   .........................................ATGCTCACATTTACCTGTATGAGCATCCTAAAGTAGAGG   39
DFR2(V4).SEQ   TGGATTTCTTCAAATGGATATTGTCAGTGCTCAGAGGGAAGAATTATGGAAAGATGAAGACGTGGCTTCCAGACTACACG  960

DFR2(V2).SEQ   AACGCTACATTTGCTCTTCT...CATGATGCCACCATTTA....CGATCTAGCTGATTTAACCAGGGGGAAATACCTGAA  112
DFR2(V4).SEQ   GAAACAACACCGGGTCTTCAGTACAAGGACTTACGAGTGGGAAGCGGCCCTGTAGTGGAAATGAGAGAGCTGGTAATGTT 1040

DFR2(V2).SEQ   TTACCATGTCCCTACTGAGTAAGAAATATCTTTTCCTTCCAATAAATTC.ATTTATGTATTAGAAATTATAAGGCTGTAA  191
DFR2(V4).SEQ   CTGCAATGCTCACATTTACCTGTATGAGCATCCTAAAGTAGAGGAACGCTACATTTGCTCTTCTCATGATGCCACCATTT 1120

DFR2(V2).SEQ   AATGTGTATTTAGATTGAGTAATGGTATGCTC....AACACAATG.....AACAGGTTCAACGACATTGGTAGGAACCTG  262
DFR2(V4).SEQ   ACGATCTAGCTGATTTAACCAGGGGGAAATACCTGAATTACCATGTCCCTACTGAGTTCAACGACATTGGTAGGAACCTG 1200

DFR2(V2).SEQ   AAGGTTGTCTCTTTCTCATCAAAGAAGTTGACAGACTTGGGGTTCCAATTCAAGTATGGAGACATGCAAAAAGGAGCCAT  342
DFR2(V4).SEQ   AAGGTTGTCTCTTTCTCATCAAAGAAGTTGACAGACTTGGGGTTCCAATTCAAGTATGGAGACATGCAAAAAGGAGCCAT 1280

DFR2(V2).SEQ   TGAGACTTGCCGTGAGAAAGAATTGATTCCTCTTTTTAGTGAGAAGGAGAAACATGCATGCCAGTGGAGAGAACTAGAGG  422
DFR2(V4).SEQ   TGAGACTTGCCGTGAGAAAGAATTGATTCCTCTTTTTAGTGAGAAGGAGAAACATGCATGCCAGTGGAGAGAACTAGAGG 1360

DFR2(V2).SEQ   AAGAAATAAATGGATTGTCTATCTTAATTTCTATTTATTTTTGGAAATAGTTGTATGGTGTGAAAGTTGCAATGGGTCAC  502
DFR2(V4).SEQ   AAGAAATAAATGGATTGTCTATCTTAATTTCTATTTATTTTTGGAAATAG.............................. 1410

DFR2(V2).SEQ   CTGCAGGATGAAAGTATGCAACTATAAGAGTATATTTTGTTTCAAACCTGTAGTCCTGACTTTGTTTATAGCTAGTGAAA  582
DFR2(V4).SEQ   ................................................................................ 1410

DFR2(V2).SEQ   CTATACAACCAACAAGTCTTCAATTTCTATAAAATTTTCTAGTGTTCCTC                                632
DFR2(V4).SEQ   ..................................................                               1410
```

Fig. S1 Sequence alignment of the coding sequence of the *DFR2* gene in different genome annotations. The gene sequences from commonly used version 2 (V2) and newly annotated version 4 (V4) grape genomes were used for alignment.

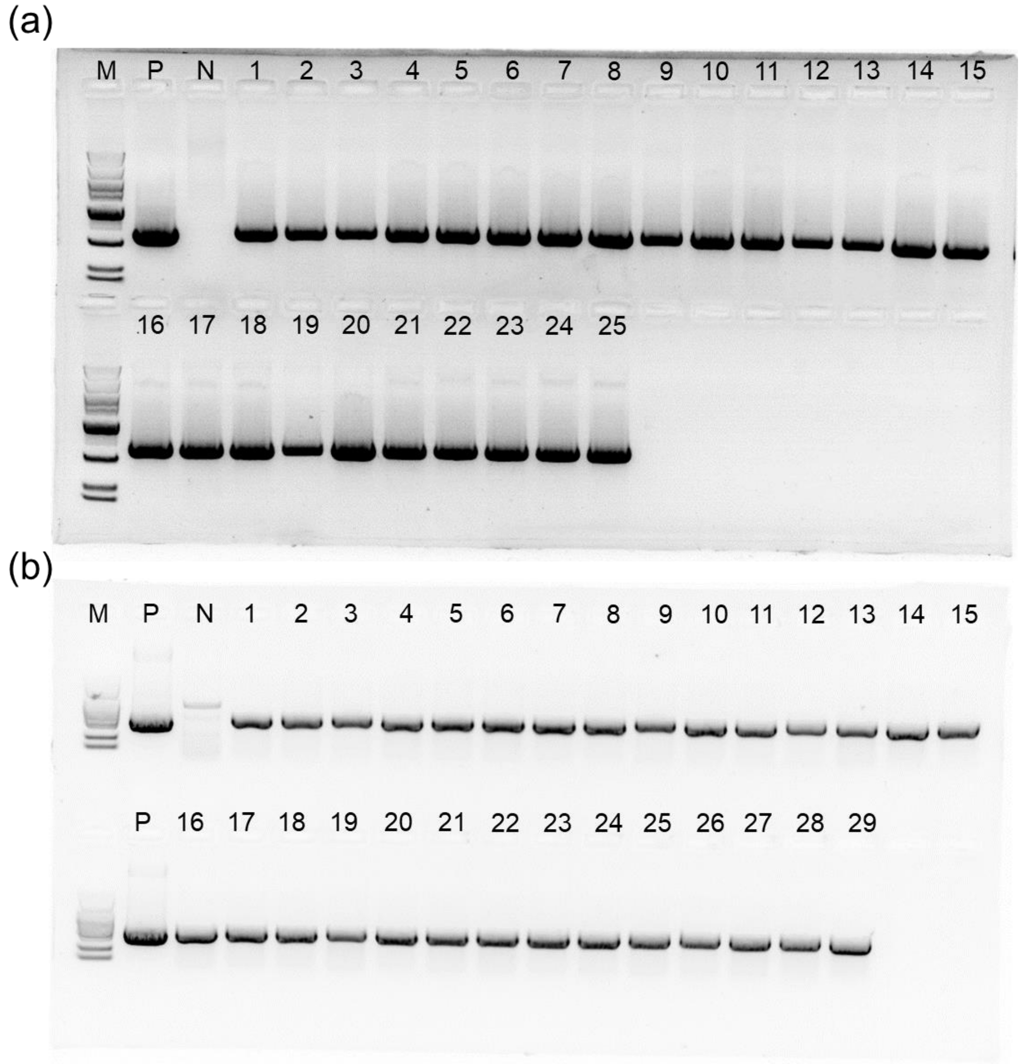

Fig. S2 PCR identification of transgenic plants regenerated from 41B embryogenic grape cells transformed with LbCas12a-TMT1 without (a) or with (b) heat treatment. The constructed plasmid and genomic DNA from wile-type plant were used as positive (P) and negative (N) controls, respectively. Lanes 1-25 in (a) and Lanes 1-29 in (b) represent independent regenerated plants.

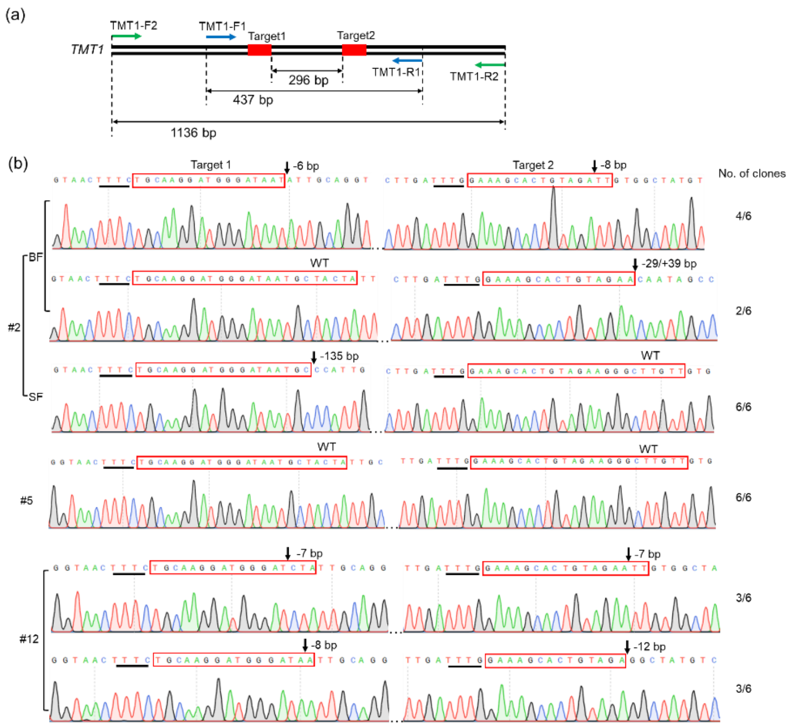

	Target 1 / Target 2	Mutation	No. of clones
#3	TAACTTTCTGCAAGGATGGGATA------CTAT/290bp/ATTTGGAAAGCACTGTAGAA-------------	-6/-13 bp	2/6
	TAACTTTCTGCAAGGATGGGATA------CTAT/290bp/ATTTGGAAAGCACTGTAGAAGGGCTTGTTGTG	-6 bp/WT	4/6
#4	TAACTTTCTGCAAGGATGGGATAATG---CTAT/290bp/ATTTGGAAAGCACTGTAGAAGGGC---TTGTG	-3/-3 bp	3/6
	TAACTTTCTGCAAGGATGGGATA------CTAT/290bp/ATTTGGAAAGCACTGTAGAAGGGC----TGTG	-6/-4 bp	3/6
#6	TAACTTTCTGCAAGGATGGGATAAT--------/290bp/ATTTGGAAAGCACTGTAGAAG------TTGTG	-8/-6 bp	1/6
	TAACTTTCTGCAAGGATGGGATA------CTAT/290bp/ATTTGGAAAGCACTGTAGAAGGGCT---TGTG	-6/-3 bp	3/6
	TAACTTTCTGCAAGGATGGGATAA-----CTAT/290bp/ATTTGGAAAGCACTGTAGAAGGGCT----GTG	-5/-4 bp	2/6
#7	TAACTTTCTGCAAGGATGGGATAA-----CTAT/290bp/ATTTGGAAAGCACTGTAGAAGGGC----TGTG	-5/-4 bp	5/6
	TAACTTTCTGCAAGGATGGGATA---CTACTAT/290bp/ATTTGGAAAGCACTGTAGAAGGGCT---TGTG	-3/-3 bp	1/6
#8	TAACTTTCTGCAAGGATGGGATAAT-------T/290bp/ATTTGGAAAGCACTGTAGAAGGGC----TGTG	-7/-4 bp	3/6
	TAACTTTCTGCAAGGATGGGATA---CTACTAT/290bp/ATTTGGAAAGCACTGTAGAAGG-----TTGTG	-3/-5 bp	3/6
#9	TAACTTTCTGCAAGGATGGGATAA-----CTAT/290bp/ATTTGGAAAGCACTGTAGAAGGGCT---TGTG	-5/-3 bp	2/6
	TAACTTTCTGCAAGGATGGGATA----TACTAT/290bp/ATTTGGAAAGCACTGTAGAAGGG-----TGTG	-4/-5 bp	4/6
#10	TAACTTTCTGCAAGGATGGGATA---------T/290bp/ATTTGGAAAGCACTGTAGAAGGGCTTGTTGTG	-9 bp/WT	2/6
	TAACTTTCTGCAAGGATGGGATA---CTACTAT/290bp/ATTTGGAAAGCACTGTAGAAG------TTGTG	-3/-6 bp	4/6
#11	TAACTTTCTGCAAGGATGGGATA---CTACTAT/290bp/ATTTGGAAAGCACTGTAGAAGGGCT----GTG	-3/-4 bp	1/6
	TAACTTTCTGCAAGGATGGGATA------CTAT/290bp/ATTTGGAAAGCACTGTAGAAGGGCTTGTTGTG	-6 bp/WT	2/6
	TAACTTTCTGCAAGGATGGGATAA----ACTAT/290bp/ATTTGGAAAGCACTGTAGAAGGGCTT---GTG	-4/-3 bp	2/6
#13	TAACTTTCTGCAAGGATGGGATAATGCTACTAT/290bp/ATTTGGAAAGCACTGTAGAAGGGCTTGTTGTG	WT/WT	6/6
#14	TAACTTTCTGCAAGGATGGGATAAT--------/290bp/ATTTGGAAAGCACTGTAGAAG------TTGTG	-8/-6 bp	2/6
	TAACTTTCTGCAAGGATGGGATAAT-------T/290bp/ATTTGGAAAGCACTGTAGAAGGG-----TGTG	-7/-5 bp	4/6
#15	TAACTTTCTGCAAGGATGGGATA------CTAT/290bp/ATTTGGAAAGCACTGTAGAAGGGCTTGTTGTG	-6 bp/WT	5/6
	TAACTTTCTGCAAGGATGGGATA---CTACTAT/290bp/ATTTGGAAAGCACTGTAGAAGG-----TTGTG	-3/-5 bp	1/6
#16	TAACTTTCTGCAAGGATGGGATAA----ACTAT/290bp/ATTTGGAAAGCACTGTAGAAGGGC---TTGTG	-4/-3bp	4/6
	TAACTTTCTGCAAGGATGGGATAATG-----AT/290bp/ATTTGGAAAGCACTGTAGAA------GTTGTG	-5/-6bp	1/6
	TAACTTTCTGCAAGGATGGGATAATG---CTAT/290bp/ATTTGGAAAGCACTGTAGAAGGGCTTGTTGTG	-3 bp/WT	1/6
#17	TAACTTTCTGCAAGGATGGGATAAT-------T/290bp/ATTTGGAAAGCACTGTAG------TTGTTGTG	-7/-6bp	3/6
	TAACTTTCTGCAAGGATGGGA--------CTAT/290bp/ATTTGGAAAGCACTGTAGAAGGG----TTGTG	-8/-4 bp	3/6
#18	TAACTTTCTGCAAGGATGGGAT---GCTACTAT/290bp/ATTTGGAAAGCACTGTAGAAG------TTGTG	-3/-6bp	5/6
	TAACTTTCTGCAAGGATGGGATAATGCTACTAT/290bp/ATTTGGAAAGCACTGTAGAAGG-----TTGTG	WT/-5 bp	1/6
#19	TAACTTTCTGCAAGGATGGGATAATGCTACTAT/290bp/ATTTGGAAAGCACTGTAGAAGGGCTTGTTGTG	WT/WT	1/6
	TAACTTTCTGCAAGGATGGGATAAT-------T/290bp/ATTTGGAAAGCACTGTAG------TTGTTGTG	-7/-6bp	4/6
	TAACTTTCTGCAAGGATGGGATA---CTACTAT/290bp/ATTTGGAAAGCACTGTAGAAGG-----TTGTG	-3/-5 bp	1/6
#20	TAACTTTCTGCAAGGATGGGATAA-----CTAT/290bp/ATTTGGAAAGCACTGTAGAAGGGCT---TGTG	-5/-3 bp	3/6
	TAACTTTCTGCAAGGATGGGATA----TACTAT/290bp/ATTTGGAAAGCACTGTAGAAGGG-----TGTG	-4/-5 bp	3/6
#21	TAACTTTCTGCAAGGATGGGATAA-----CTAT/290bp/ATTTGGAAAGCACTGTAGAAGG-----TTGTG	-5/-5 bp	1/6
	TAACTTTCTGCAAGGATGGGATA-------TAT/290bp/ATTTGGAAAGCACTGTAGAAGGG-----TGTG	-7/-5 bp	5/6
#22	TAACTTTCTGCAAGGATGGGATA------CTAT/290bp/ATTTGGAAAGCACTGTAGAAGGGC----TGTG	-6/-4 bp	3/6
	TAACTTTCTGCAAGGATGGGATAA-----CTAT/290bp/ATTTGGAAAGCACTGTAGAAGGGCT---TGTG	-5/-3 bp	3/6
#23	TAACTTTCTGCAAGGATGGGATA------CTAT/290bp/ATTTGGAAAGCACTGTAGAAGGGC----TGTG	-6/-4 bp	4/6
	TAACTTTCTGCAAGGATGGGATAATG-----AT/290bp/ATTTGGAAAGCACTGTAG------TTGTTGTG	-5/-6 bp	2/6
#24	TAACTTTCTGCAAGGATGGGATA---------T/290bp/ATTTGGAAAGCACTGTAGAA-------TTGTG	-9/-7 bp	2/6
	TAACTTTCTGCAAGGATGGGATAATG----TAT/290bp/ATTTGGAAAGCACTGTAGAAGGGCTTGTTGTG	-4 bp/WT	4/6
#26	TAACTTTCTGCAAGGATGGGATA---CTACTAT/290bp/ATTTGGAAAGCACTGTAGAAGGGCTTGTTGTG	-3 bp/WT	2/6
	TAACTTTCTGCAAGGATGGGATAA-----CTAT/290bp/ATTTGGAAAGCACTGTAGAAGG----GTTGTG	-5/-4 bp	2/6
	TAACTTTCTGCAAGGATGGGATA---------T/290bp/ATTTGGAAAGCACTGTAGAAGG-----TTGTG	-9/-5 bp	1/6
	TAACTTTCTGCAAGGATGGGAT--------TAT/290bp/ATTTGGAAAGCACTGTAGAAGGGCTTGTTGTG	-8 bp/WT	1/6
#27	TAACTTTCTGCAAGGATGGGATAAT--------/290bp/ATTTGGAAAGCACTGTAGAAGGGCTTGTTGTG	-8 bp/WT	1/6
	TAACTTTCTGCAAGGATGGGATA-------TAT/290bp/ATTTGGAAAGCACTGTAGAAGGG----TTGTG	-7/-4 bp	1/6
	TAACTTTCTGCAAGGATGGGATAAT------AT/290bp/ATTTGGAAAGCACTGTAGAAGGGC-----GTG	-6/-5 bp	3/6
	TAACTTTCTGCAAGGATGGGAT---------AT/290bp/ATTTGGAAAGCACTGTAGAAGGGCTTGTTGTG	-9 bp/WT	1/6
#28	TAACTTTCTGCAAGGATGGGATA---CTACTAT/290bp/ATTTGGAAAGCACTGTAGAAGG-----TTGTG	-3/-5bp	5/6
	TAACTTTCTGCAAGGATGGGATAA----ACTAT/290bp/ATTTGGAAAGCACTGTAGAAGGGTCCGCCTCA	-4/-14,+1 bp	1/6
#29	TAACTTTCTGCAAGGATGGGATAATG--ACTAT/290bp/ATTTGGAAAGCACTGTAGAA--------TGTG	-2/-8 bp	2/6
	TAACTTTCTGCAAGGATGGGAT-----------/290bp/ATTTGGAAAGCACTGTAGAAGGG----TTGTG	-11/-4 bp	1/6
	TAACTTTCTGCAAGGATGGGATAA-----CTAT/290bp/ATTTGGAAAGCACTGTAG----------TGTG	-5/-10 bp	2/6
	TAACTTTCTGCAAGGATGGGATAA----ACTAT/290bp/ATTTGGAAAGCACTGTAGAAG-------TGTG	-4/-7 bp	1/6

Fig. S3 Sequencing results of *TMT1* editing in LbCas12a-TMT1 grapevine plants. The results obtained with transgenic plants after HT are shown as examples. (a) Schematic illustration of primers design for amplification of target sequences of *TMT1* gene. (b) Sanger sequencing results of the two targets in grapevine plants. Representative sequencing chromatograms as well as identified sequences are shown. The PAM sequences are underlined and the targets are shown in red box in chromatograms. For each plant, 6 clones of amplicons were randomly selected and analyzed by Sanger sequencing. Mutation types and corresponding number of clones are shown on the right.

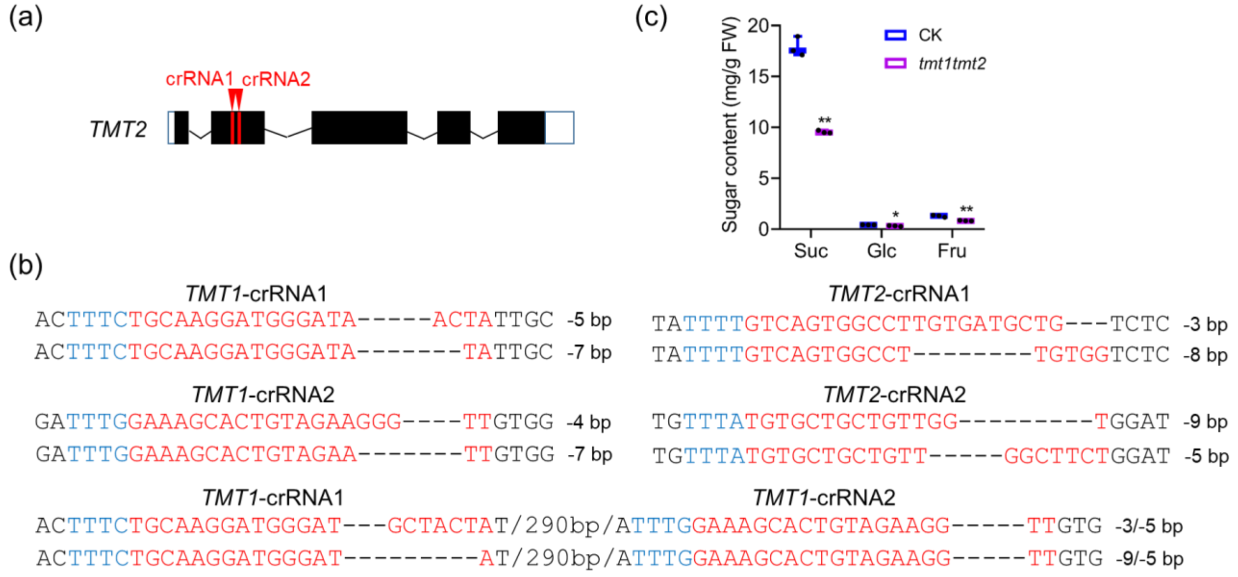

Fig. S4 Knockout of *TMT1* and *TMT2* genes in grape. (a) Schematic illustration of the targets design for *TMT2*. (b) Representative mutated sequences detected at the four targets in *TMT1* and *TMT2*. (c) Sugar content in *tmt1tmt2* cells. The cells transformed with empty vector were used as the control (CK). The significant differences are determined by Student's *t*-test. * $P < 0.05$; ** $P < 0.01$. Suc, sucrose; Glc, glucose, Fru, fructose.

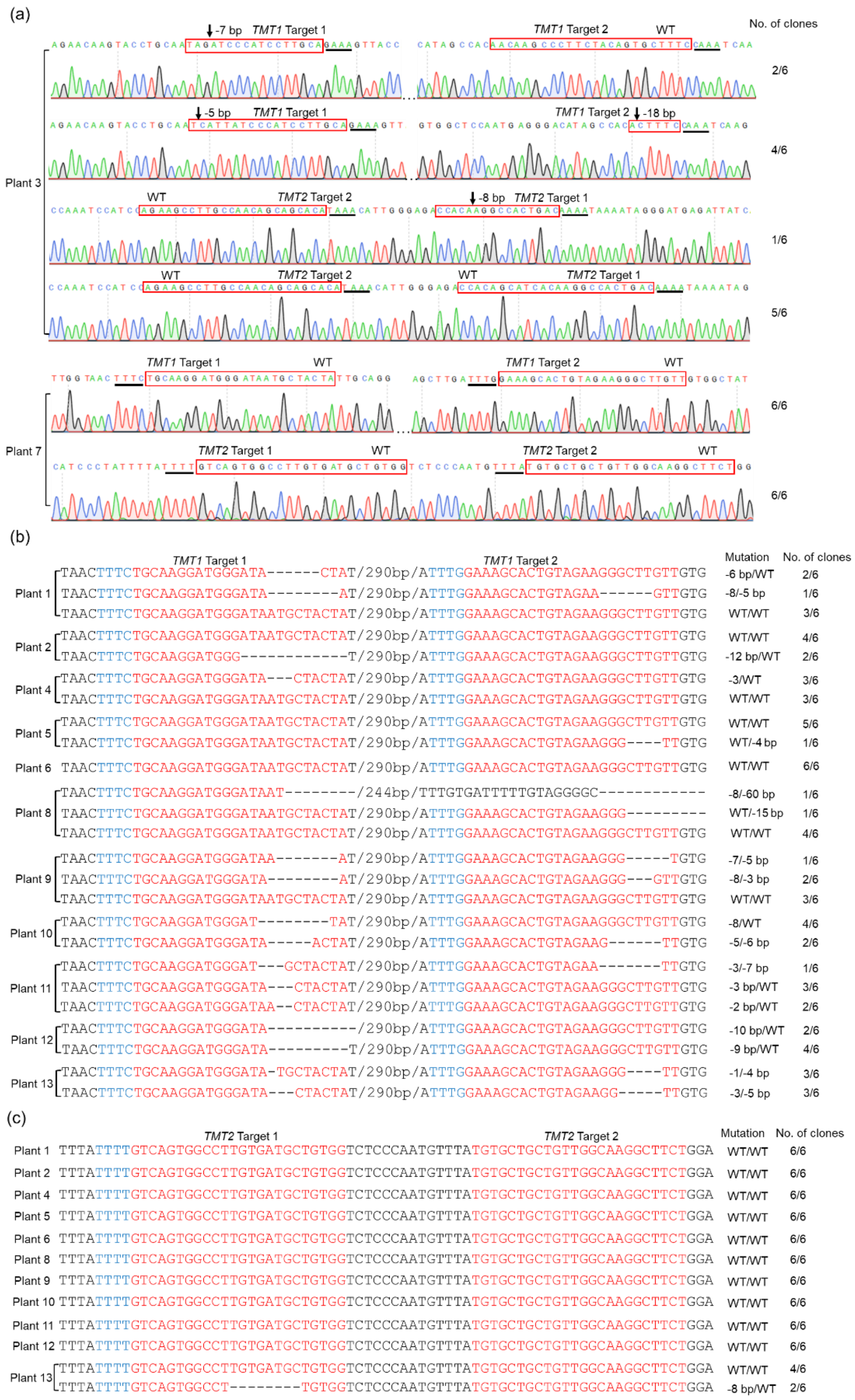

Fig. S5 Sequencing results of *TMT1* and *TMT2* editing in LbCas12a-TMTs plants. Representative sequencing chromatograms (a) and identified sequences for each plant (b and c) are shown. The PAM sequences are underlined and the targets are shown in red box in chromatograms. For each plant, 6 clones of amplicons were randomly selected and analyzed by Sanger sequencing. Mutation types and corresponding number of clones are shown on the right.

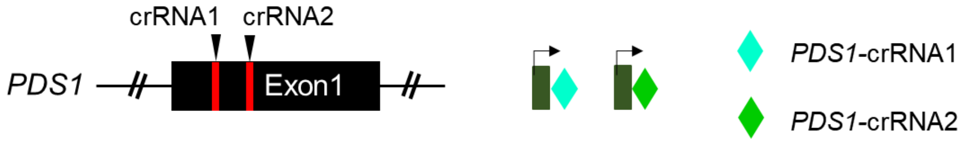

Fig. S6 Schematic illustration of the targets design for *PDS1*.

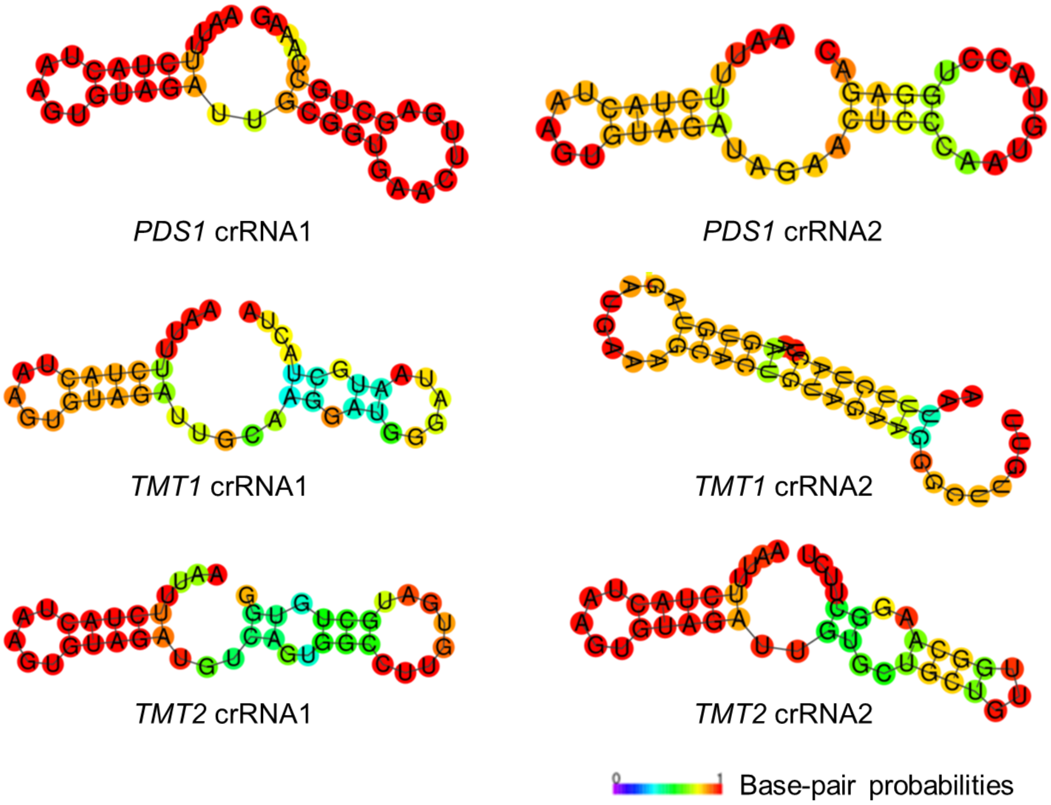

Fig. S7 Predication of the secondary structures of *PDS1* and *TMTs* crRNAs. The RNA secondary structures were predicted using *RNAfold* web server (http://rna.tbi.univie.ac.at/cgi-bin/RNAWebSuite/RNAfold.cgi) with default parameters.

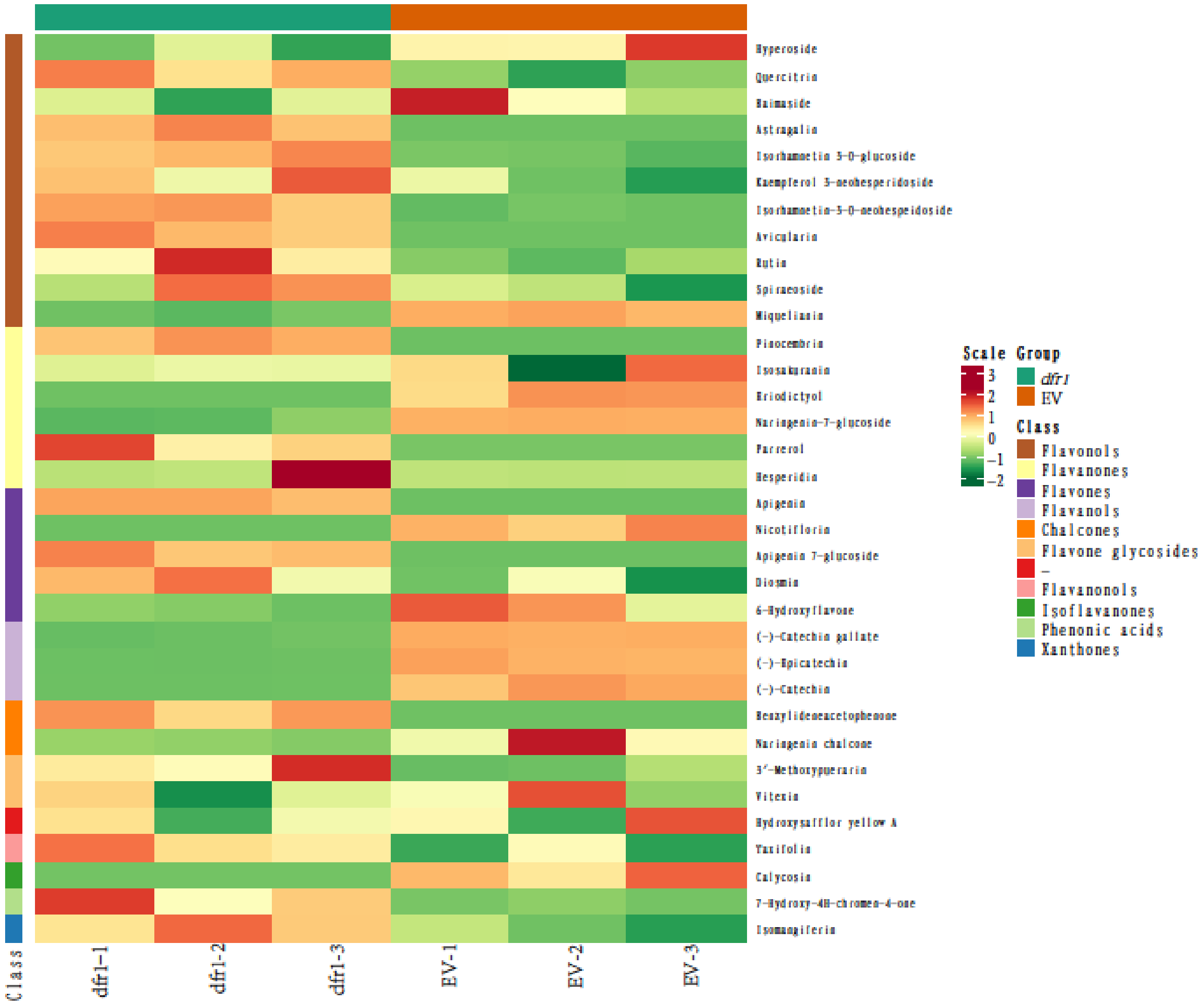

Fig. S8 Heatmap of the determined compounds in *dfr1* and EV cells. The log2-transformed values were visualized as the heatmap using *R*.

VvU3.1-*TMT1* crRNAs:

AAGCTTAGTACTTTCATAGGAATAGGTTTCAAATGAACCTTTGTGATACACTTCGATCCTGACTCTCTCTAAAAGCAAA
AATCATTAATATTTTTTCAATTATATTTTAATTTTTACAAATACTAACAAATATTAATAATTCTAATATCTTTCTTGTT
TTGAAAAATAAAAGAAAAATAATAATGTTTGGTATATTCCGTATATATTATTTTAAATGAATCCAGAAGTTTCCAAGAA
TTTCACTGGCAATCAATCGTGCATCAGCTGTCAATCGTTGTTCCCAGGAAGGCTCATTGGAAGTCTATAACCAATGAGA
ACACGCGGTGACTAGCCGTCCCACATCGAAAATGCAGGAAACATTTAATAACTATATAACAAAGGATAGGAGATTCACA
TGCCAATTTCTACTAAGTGTAGATTGCAAGGATGGGATAATGCTACTAAATTTCTACTAAGTGTAGATGAAAGCACTGT
AGAAGGGCTTGTTTTTTTTAAGCTT

VvU3.1-*TMTs* crRNAs:

AAGCTTAGTACTTTCATAGGAATAGGTTTCAAATGAACCTTTGTGATACACTTCGATCCTGACTCTCTCTAAAAGCAAA
AATCATTAATATTTTTTCAATTATATTTTAATTTTTACAAATACTAACAAATATTAATAATTCTAATATCTTTCTTGTT
TTGAAAAATAAAAGAAAAATAATAATGTTTGGTATATTCCGTATATATTATTTTAAATGAATCCAGAAGTTTCCAAGAA
TTTCACTGGCAATCAATCGTGCATCAGCTGTCAATCGTTGTTCCCAGGAAGGCTCATTGGAAGTCTATAACCAATGAGA
ACACGCGGTGACTAGCCGTCCCACATCGAAAATGCAGGAAACATTTAATAACTATATAACAAAGGATAGGAGATTCACA
TGCCAATTTCTACTAAGTGTAGATTGCAAGGATGGGATAATGCTACTAAATTTCTACTAAGTGTAGATGAAAGCACTGT
AGAAGGGCTTGTTAATTTCTACTAAGTGTAGATGTCAGTGGCCTTGTGATGCTGTGGAATTTCTACTAAGTGTAGATTG
TGCTGCTGTTGGCAAGGCTTCTTTTTTTAAGCTT

The reverse *TMTs*-crRNAs:

AAGCTTAGTACTTTCATAGGAATAGGTTTCAAATGAACCTTTGTGATACACTTCGATCCTGACTCTCTCTAAAAGCAAA
AATCATTAATATTTTTTCAATTATATTTTAATTTTTACAAATACTAACAAATATTAATAATTCTAATATCTTTCTTGTT
TTGAAAAATAAAAGAAAAATAATAATGTTTGGTATATTCCGTATATATTATTTTAAATGAATCCAGAAGTTTCCAAGAA
TTTCACTGGCAATCAATCGTGCATCAGCTGTCAATCGTTGTTCCCAGGAAGGCTCATTGGAAGTCTATAACCAATGAGA
ACACGCGGTGACTAGCCGTCCCACATCGAAAATGCAGGAAACATTTAATAACTATATAACAAAGGATAGGAGATTCACA
TGCCAATTTCTACTAAGTGTAGATTGTGCTGCTGTTGGCAAGGCTTCTAATTTCTACTAAGTGTAGATGTCAGTGGCCT
TGTGATGCTGTGGAATTTCTACTAAGTGTAGATGAAAGCACTGTAGAAGGGCTTGTTAATTTCTACTAAGTGTAGATTG
CAAGGATGGGATAATGCTACTA
TTTTTTAAGCTT

The reverse *PDS1*-*TMTs*-crRNAs:

AAGCTTAGTACTTTCATAGGAATAGGTTTCAAATGAACCTTTGTGATACACTTCGATCCTGACTCTCTCTAAAAGCAAA
AATCATTAATATTTTTTCAATTATATTTTAATTTTTACAAATACTAACAAATATTAATAATTCTAATATCTTTCTTGTT
TTGAAAAATAAAAGAAAAATAATAATGTTTGGTATATTCCGTATATATTATTTTAAATGAATCCAGAAGTTTCCAAGAA
TTTCACTGGCAATCAATCGTGCATCAGCTGTCAATCGTTGTTCCCAGGAAGGCTCATTGGAAGTCTATAACCAATGAGA
ACACGCGGTGACTAGCCGTCCCACATCGAAAATGCAGGAAACATTTAATAACTATATAACAAAGGATAGGAGATTCACA
TGCCAATTTCTACTAAGTGTAGATTGCGGTGAACTTGAGCTGCCAAAGAATTTCTACTAAGTGTAGATAGAACTCCCAA
TGTACCTGGAGACAATTTCTACTAAGTGTAGATTGTGCTGCTGTTGGCAAGGCTTCTAATTTCTACTAAGTGTAGATGT
CAGTGGCCTTGTGATGCTGTGGAATTTCTACTAAGTGTAGATGAAAGCACTGTAGAAGGGCTTGTTAATTTCTACTAAG
TGTAGATTGCAAGGATGGGATAATGCTACTATTTTTTAAGCTT

Fig. S9 Sequences of *TMT1*, *TMTs* (*TMT1* and *TMT2*), reverse *TMTs* and reverse *PDS1*-*TMTs* crRNAs expression cassettes. The VvU3.1 promoter is indicated in blue and DR sequences are denoted in red. The two designed *TMT1* crRNA targets are indicated in green and purple, and the two crRNA targets for *TMT2* are indicated in orange and light

blue, respectively. The two crRNA targets for *PDS1* are indicated in black and pink, respectively. The *Hin*dIII recognition sites are highlighted in yellow.

VvU3. 1-*DFR1* crRNAs:

AAGCTTAGTACTTTCATAGGAATAGGTTTCAAATGAACCTTTGTGATACACTTCGATCCTGACTCTCTCTAAAAGCAAA
AATCATTAATATTTTTTCAATTATATTTTAATTTTTACAAATACTAACAAATATTAATAATTCTAATATCTTTCTTGTT
TTGAAAAATAAAAGAAAAATAATAATGTTTGGTATATTCCGTATATATTATTTTAAATGAATCCAGAAGTTTCCAAGAA
TTTCACTGGCAATCAATCGTGCATCAGCTGTCAATCGTTGTTCCCAGGAAGGCTCATTGGAAGTCTATAACCAATGAGA
ACACGCGGTGACTAGCCGTCCCACATCGAAAATGCAGGAAACATTTAATAACTATATAACAAAGGATAGGAGATTCACA
TGCCAATTTCTACTAAGTGTAGATTTGCTTCCTCGCCGATTCCAGGATAATTTCTACTAAGTGTAGATATCGGTTCATG
GCTGGTCATGAGGTTTTTTAAGCTT

Fig. S10 Sequence of *DFR1* crRNAs expression cassette. The VvU3.1 promoter is indicated in blue and DR sequences are denoted in red. The two designed crRNA targets for *DFR1* are underlined. The *Hin*dIII recognition sites are highlighted in yellow.
